# P(O)R_2_-directed Pd-catalyzed C–H functionalization of biaryl derivatives to synthesize chiral phosphorous ligands

**DOI:** 10.3762/bjoc.10.215

**Published:** 2014-09-02

**Authors:** Rong-Bin Hu, Hong-Li Wang, Hong-Yu Zhang, Heng Zhang, Yan-Na Ma, Shang-dong Yang

**Affiliations:** 1 State Key Laboratory of Applied Organic Chemistry, Lanzhou University, Lanzhou 730000, P. R. China; 2 State Key Laboratory for Oxo Synthesis and Selective Oxidation, Lanzhou Institute of Chemical Physics, Lanzhou 730000, P. R. China

**Keywords:** chiral synthesis, Pd-catalysis, organophosphorus, phosphorus ligands, P(O)R_2_-directing

## Abstract

Chiral phosphorus ligands have been widely used in transition metal-catalyzed asymmetric reactions. Herein, we report a new synthesis approach of chiral biaryls containing a phosphorus moiety using P(O)R_2_-directed Pd-catalyzed C–H activation; the functionalized products are produced with good enantioselectivity.

## Introduction

In the past decades, phosphorus ligands have been demonstrated to be efficient ligands in many metal-catalyzed organic reactions [1–4]. In particular, their special effects of enhancing the metal-catalyst efficiency and of controlling chiral induction has continually prompted synthetic chemists to probe efficient methods generating access to chiral, enantiomerically pure phosphorus compounds used in pharmaceutical, agrochemical and perfume industries [5–10]. However, the difficulty of synthesizing such ligands hampered their wide application, mainly due to the challenging formation of P–X (X = N, O, C…), especially C–P bond formation.

**Scheme 1:**
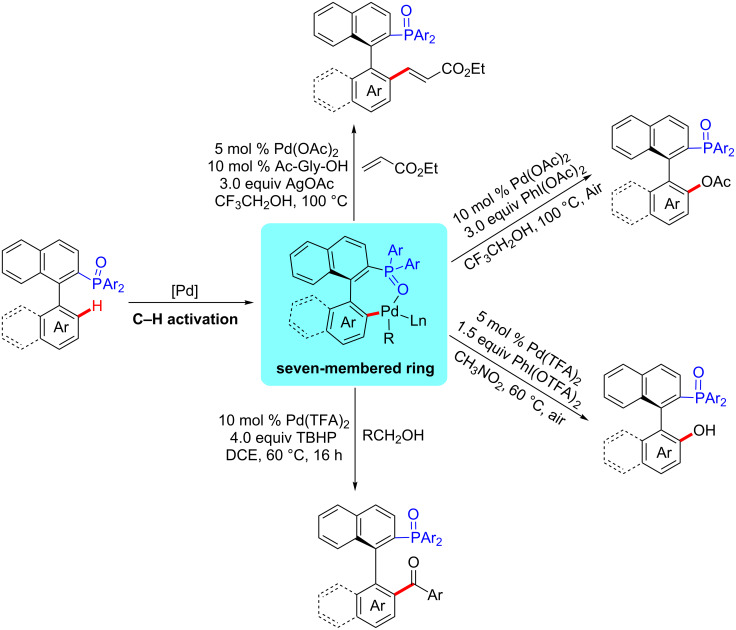
C–H functionalization of P(O)R_2_ directed through a seven-membered cyclopalladium transition state.

At present, the traditional strategy to introduce phosphorus atoms requires prefunctionalization or lithiation of substrate. However, these methods are not compatible for some activated functional groups in precursor compounds. Over the past several years, we have achieved reactions of C–P bond formation with new and efficient protocols via transition metal-catalysis [11–16]. Despite the progress in this area, only limited development has been accomplished through metal-catalyzed C–H activation to build C–P bonds [17–18]. As an alternative, we disclosed a novel protocol of palladium-catalyzed C–H functionalization by using the P(O)R_2_ moiety as a new directing group to synthesize a series of phosphorus-containing compounds in a straightforward and atom-efficient way (Scheme 1) [19–23]. In our system, we proposed a seven-membered cyclopalladium transition state instead of the common five or six-membered transition state [24–30]. The P(O)R_2_ group not only achieved the directing role but also acts as an important component unit of the C–H functionalized products. In this paper, we use the axially chiral biaryl phosphine oxides as substrates and report the synthesis of various chiral phosphorus ligands with high enantiomeric selectivity using palladium-catalyzed C–H functionalization.

## Results and Discussion

**Scheme 2:**
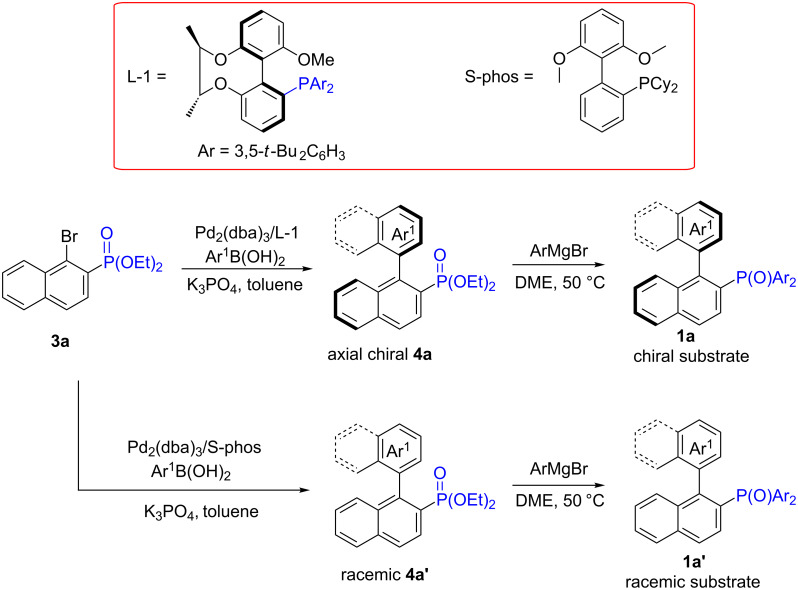
Synthesis of chiral and racemic substrates.

To obtain the axially chiral phosphorus compounds, we first synthesized the special chiral-bridged atropisomeric monophosphorus ligand L-1 through an eight-step reaction sequence starting from 1,3-dimethoxybenzene. According to the reported operation, the substrates of biaryl derivatives that contained phosphate with axial chirality were obtained in high yields using the Suzuki–Miyaura coupling reaction with the assistance of this versatile chiral ligand [31–34]. We used substituted naphthylboronic acid or *ortho*-substituted-phenylboronic acid to synthesize the corresponding substituted binaphthyl or phenyl-naphthyl skeleton substrates with axial chirality. To maintain the axial chirality within the substrates, a steric hindrance effect at the ortho position of phenylboronic acids was required, rendering the *non-ortho* substituted-phenylboronic acid that is not applicable in these reactions. As the P(O)Ar_2_ group showed a better directing ability in the process of C–H activation, the axial chiral P(O)(OEt)_2 _**4a** was transformed into P(O)Ar_2_ by reacting with an arylgrignard reagent (Scheme 2) [32]. At the same time, the racemic substrates were produced using the non-chiral S-phos ligand. By using 2-chlorophenylboronic acid as coupling component, we demonstrated that we could obtain the phosphate compound, but it failed to yield the P(O)Ph_2_ group in the arylation step. In addition, in the processes of hydroxylation, arylation, alkenylation, the P(O)(iPr)_2_ group showed a good guiding ability, but the corresponding substrates could not be obtained because the phosphate moiety did not react with the (iPr)MgBr.

**Figure 1:**
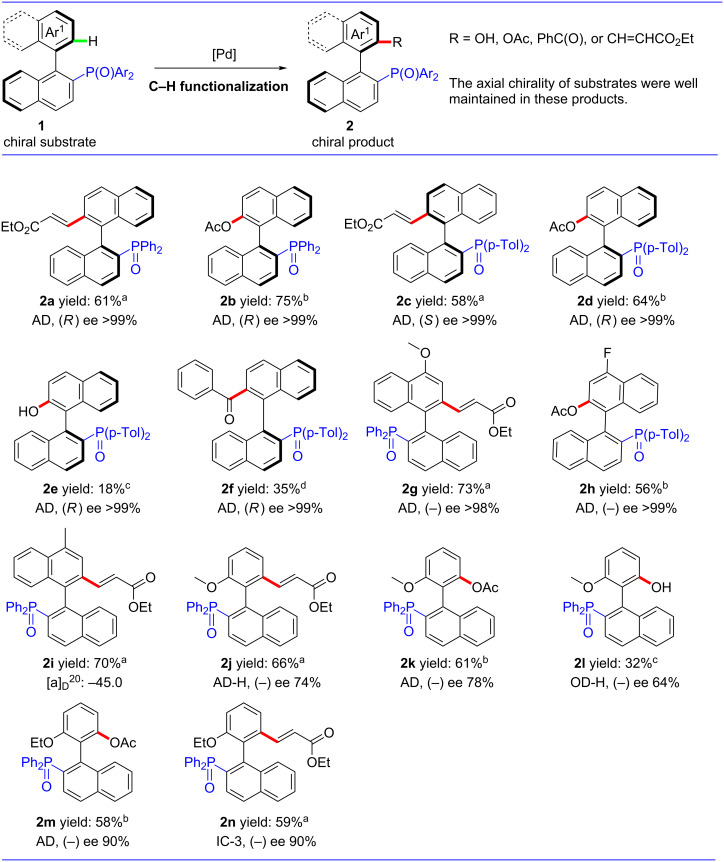
C–H functionalization of axially chiral phosphorus substrates. The yields are isolated yields and the ee values are determind by HPLC. ^a^Reaction conditions: substrate (0.3 mmol), ethyl acrylate (1.5 mmol), Pd(OAc)_2_ (10 mol %), Ac-Gly-OH (20 mol %), AgOAc (1.5 mmol), TFE (3.0 mL), 100 °C, 24 h, air atmosphere; ^b^Substrate (0.3 mmol), PhI(OAc)_2_ (0.9 mmol), Pd(OAc)_2_ (10 mol %), TFE (3.0 mL), 100 °C, 24 h, air atmosphere; ^c^Substrate (0.3 mmol), TBHP (1.2 mmol), benzyl alcohol (0.75 mmol), Pd(TFA)_2_ (10 mol %), DCE (3.0 mL), 60 °C, air atmosphere; ^d^Substrate (0.3 mmol), PhI(TFA)_2_ (0.45 mmol), Pd(OAc)_2_ (10 mol %), MeNO_2_ (3.0 mL), 60 °C, 24 h, air atmosphere.

Under the optimized conditions, we started to investigate the scope and applicability of our strategies. Initially, we used chiral [1,1'-binaphthalen]-2-yldiphenylphosphine oxide as a substrate [35]. In the process of alkenylation and acetoxylation, the corresponding products **2a** and **2b** were obtained in moderate yields and high enantioselectivities. Next, we examined the substituent effect with P(O)(*p-*Tol)_2_ as a directing group: The reactions of alkenylation, acetoxylation, hydroxylation and acylation occurred smoothly. Even if the products were obtained in low to moderate yields, they were optically pure (Figure 1, **2c–f**). For the substrate of 4-methoxy substituted binaphthyl, we could achieve the alkenylation product **2g** in moderate yield and with high ee. When a fluorine substituent was used, the acetoxylated product **2h** was obtained in moderate yield and high ee. Even if the alkenylation product **2i** was obtained when the substituent was methyl, we failed to produce the desired chromatogram; however, it did exhibit a good optical rotation. Those results showed that the products of C–H functionalization were maintained with high enantioselectivities when the substrates were optically pure, even when these reactions were carried out in air atmosphere and at high temperature. Herein, we provided a method to synthesize the substituted axially chiral binaphthyl compounds with a phosphorus moiety. Moreover, these products can be further transformed into other functional groups.

Next, the substrates of the phenyl-naphthyl framework were examined. For the ortho-OMe substituted substrate, we achieved the products of alkenylation, acetoxylation and hydroxylation. The OMe group is a relatively small group, so the ee was not very high. If the substituent was OEt, the products of alkenylation and acetoxylation (Figure 1, **2m** and **2n**) were obtained in moderate yield and the results showed good enantioselectivities. Although the yields were not very high in these processes, the starting materials were completely converted except for the acylation reaction, presumably due to partial decomposition of the starting materials. These functionalized products showed that the axially chiral substrates could be well maintained in our system of P(O)R_2_-directed Pd-catalyzed C–H activation. These compounds could be transformed to trivalent phosphorus compounds by silane to obtain the corresponding phosphorous ligands.

## Conclusion

In summary, a series of substrates with axially chiral biaryl compounds containing a P(O)Ar_2_ directing group were successfully synthesized using the Suzuki–Miyaura coupling reaction under the assistance of a chiral ligand. Moreover, the substrates were further C–H functionalized using the P(O)Ar_2_ directing role with Pd salt as catalyst. Notably, the reactions took place in air atmosphere and at high temperature and the corresponding functionalized products exhibited good enantioselectivities. We propose a unique seven-membered cyclopalladium transition state for this transformation and provide a new and efficient route to synthesize the substituted axially-chiral oxygen–phosphine or alkene–phosphine ligand analogues.

## Experimental

See Supporting Information File 1.

## Supporting Information

File 1 Experimental details, characterization data (^1^H, ^13^C, ^31^P spectra) of products.
