# Supplementary material for: P(O)R2-directed Pd-catalyzed C–H functionalization of biaryl derivatives to synthesize chiral phosphorous ligands
Source: Beilstein J Org Chem. 2014 Sep 2;10:2071–6. doi: 10.3762/bjoc.10.215 (PMC4168959; doi:10.3762/bjoc.10.215)
Supplement: File 1 — Experimental details, characterization data (1H, 13C, 31P spectra) of products. [file Beilstein_J_Org_Chem-10-2071-s001.pdf]

Supporting Information

for

P(O)R_2-directed Pd-catalyzed C–H functionalization of biaryl derivatives to synthesize chiral phosphorous ligands

Rong-Bin Hu,[1] Hong-Li Wang,[1] Hong-Yu Zhang,[1] Heng Zhang,[1] Yan-Na Ma[1] and Shang-dong Yang*[1, 2]

Address: [1]State Key Laboratory of Applied Organic Chemistry, Lanzhou University, Lanzhou 730000, P. R. China and [2]State Key Laboratory for Oxo Synthesis and Selective Oxidation, Lanzhou Institute of Chemical Physics, Lanzhou 730000, P. R. China

Email: Shang-dong Yang - yangshd@lzu.edu.cn

* Corresponding author

Experimental details, characterization data (^{1}H, ^{13}C, ^{31}P spectra) of products

Table of Contents

Ⅰ. General methods and materials S2

Ⅱ. The synthesis route of chiral ligand L-1 and substrates S2–S3

Ⅲ. General procedures for the preparation of C–H functionalization S3–S4

Ⅳ. Characterization of the products S5–S10

Ⅴ. Copies of NMR and HPLC spectra charts S11–S45

Ⅰ. General methods and materials

^{1}H and ^{13}C NMR spectra were recorded on a Bruker advance Ⅲ 400 spectrometer (400 MHz for ^{1}H and 100 MHz for ^{13}C) in $CDCl_3$ with TMS as internal standard. Chemical shifts (δ) were measured in ppm relative to TMS δ = 0 for ^{1}H, or to chloroform δ = 77.0 for ^{13}C as internal standard. ^{31}P NMR spectra and ^{19}F NMR were recorded on the same instrument. Data are reported as follows: Chemical shift, multiplicity (s = singlet, d = doublet, dd = doublet of doublets, t = triplet, q = quartet, m = multiplet), Coupling constants, *J*, are reported in hertz. Mass data were measured with Thermo Scientific DSQ II mass spectrometer. The starting materials were purchased from Aldrich, Acros Organics, J&K Chemicals or TCI and used without further purification. Solvents were dried and purified according to the procedure from "Purification of Laboratory Chemicals book". Thin-layer chromatography (TLC) was performed using 60 mesh silica gel plates visualized with short-wavelength UV light (254 nm). Substrates were prepared according to corresponding literature. Enantioselectivities were determined by high performance liquid chromatography (HPLC, Waters-600-2996 or Agilent 1260) analysis employing a chiral column. Compound 2a was described previously in Org. Lett. 2013, 15, 5302-5305, Compound **2b** was described previously in Chem. Commun., 2014, 50, 4686-4689.

Ⅱ. The synthesis route of chiral ligand L-1 and substrates

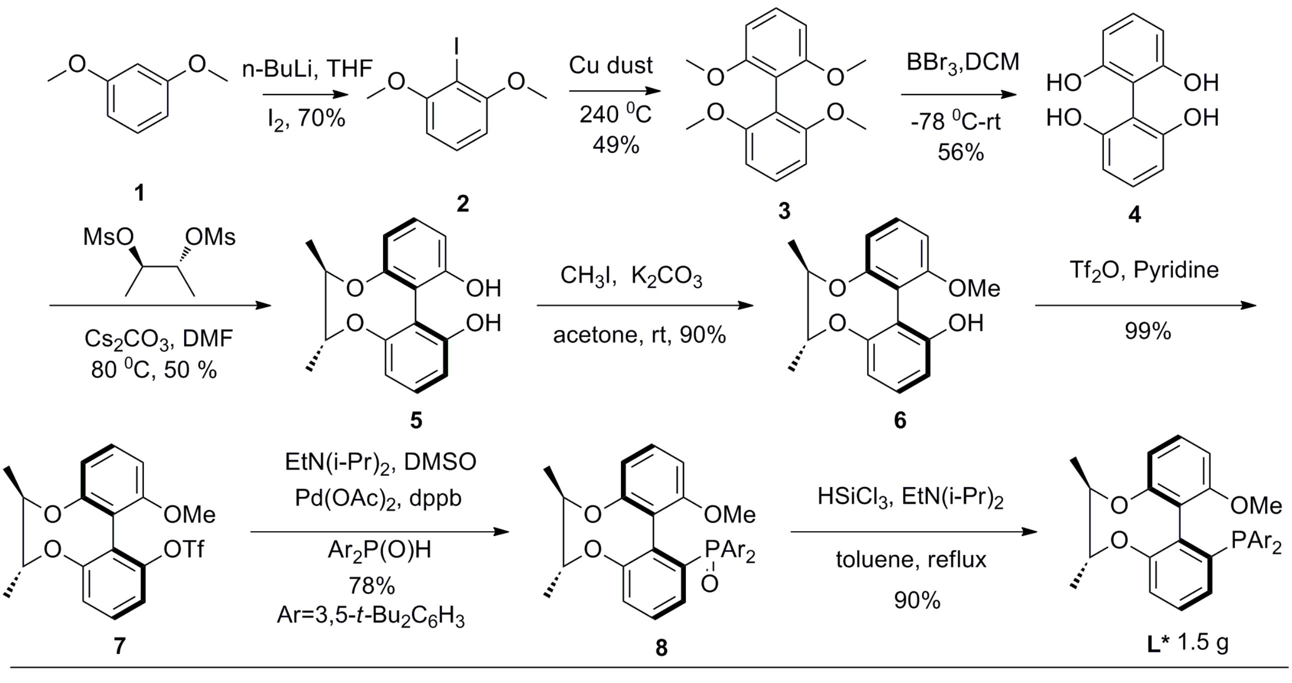

Detailed operations see following references: 1. Govender, S.; Mmutlane, E. M.; van Otterlo, W. A. L.; de Koning, C. B. *Org. Biomol. C hem.*, **2007**, *5*, 2433–2440, doi: 10.1039/b707187f; 2. Kakei, H.; Tsuji, R.; Ohshima, T.; Morimoto, H.; Matsunaga, S.; Shibasaki, M. *Chem. Asian J.*, **2007**, *2*, 257–264, doi: 10.1002/asia.200600309; 3. Wang, S. L.; Li, J. J.; Miao, T. T.; Wu, W. H.; Li, Q.; Zhuang, Y.; Zhou, Z. Y.; Qiu, L. Q. *Org. Lett.*, **2012**, *14*, 1966-1969, doi: 10.1021/ol300721p.

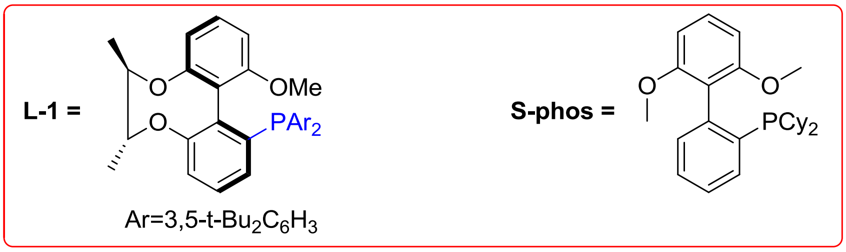

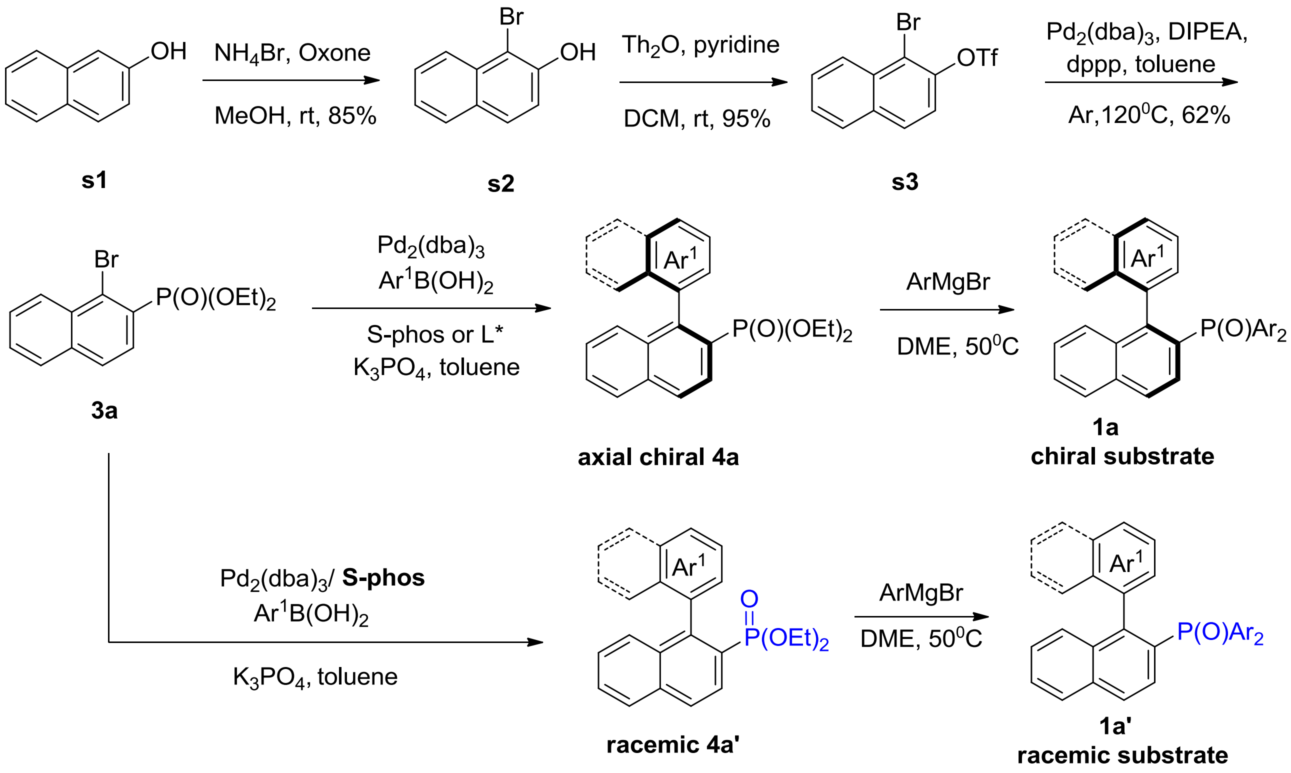

Detailed operations see following reference: Yin, J.; Buchwald, S. L. *J. Am. Chem. Soc.*, **2000**, *122*, 12051-12052, doi: 10.1021/ja005622z. 2. Wang, S. L.; Li, J. J.; Miao, T. T.; Wu, W. H.; Li, Q.; Zhuang, Y.; Zhou, Z. Y.; Qiu, L. Q. *Org. Lett.*, **2012**, *14*, 1966-1969, doi: 10.1021/ol300721p.

Ⅲ. General procedures for the preparation of C–H functionalization

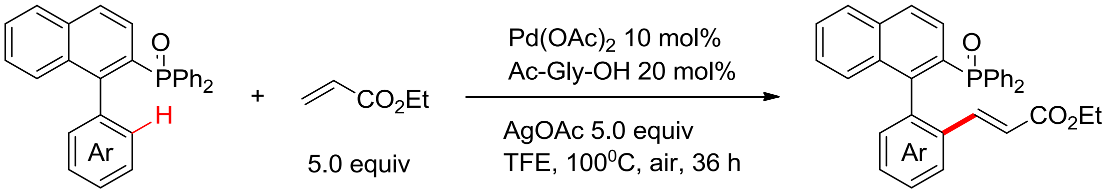

In a similar manner as described before [1]: Under air atmosphere, diarylphosphine oxide (0.30 mmol, 1 equiv), $Pd(OAc)_2$ (6.7 mg, 0.03 mmol, 10.0 mol %), Ac-Gly-OH (7.2 mg, 0.06 mmol, 20.0 mol %) and AgOAc (250.5 mg, 1.5 mmol, 5.0 equiv) were added to oven-dried reaction tube containing a magnetic stir bar. After sealed tube, a solution of ethyl acrylate (150.0, 1.5 mmol, 5.0 equiv) in 3.0 mL CF_3CH_2OH was added using a syringe. The mixture was stirred at 100℃ in an oil bath until substrate disappeared as judged by TLC. After cooling to room temperature, the solution was removed in vacuo to yield a residue, which was purified by silica gel using (1:1 EtOAc/hexane) to afford the pure product as a oil.

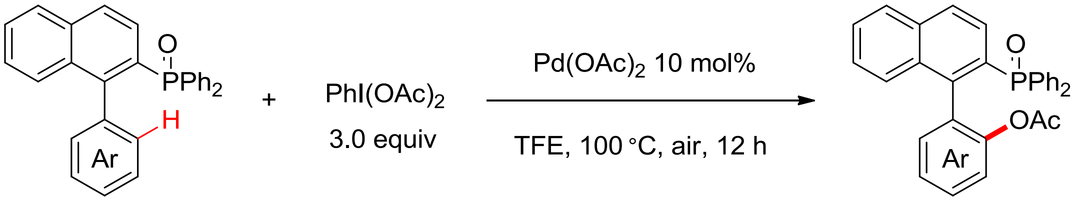

In a similar manner as described before [2]: Under air atmosphere, diarylphosphine oxide (0.30 mmol, 1.0 equiv), Pd(OAc)2 (6.7 mg, 0.03mmol, 10.0 mol %) and $PhI(OAc)_2$ (289 mg, 0.90 mmol, 3.0 equiv) were added to tube containing a magnetic stir bar. After sealed tube, 3.0 mL CF_3CH_2OH was added using a syringe. The mixture was stirred at 100 ℃ in an oil bath until substrate disappeared as judged by TLC. After cooling to room temperature, the solution was removed in vacuo to yield a residue, which was purified by silica gel using (1:1 EtOAc/hexane) to afford the pure product as an oil.

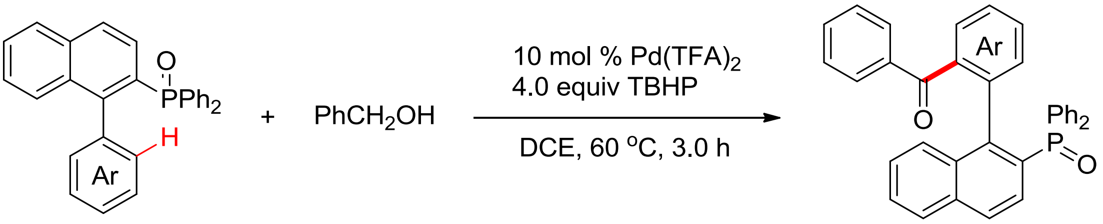

In a similar manner as described before [3]: Under air atmosphere, diarylphosphine oxide (0.20 mmol, 1.0 equiv) and $Pd(TFA)_2$ (6.6 mg, 0.02 mmol, 10 mol %) were added to a tube containing a magnetic stir bar. After which, 1.0 mL DCE was added using a syringe. Then 70 % aq. TBHP solution (110 μL, 0.80 mmol, 4.0 equiv) and benzyl alcohol 2'a (52 uL, 0.5 mmol, 2.5 equiv) were added with microsyringes. The reaction mixture was stirred at 60 °C in an oil bath for 20 hours until substrate disappeared as judged by TLC. After cooling to room temperatur, the solution was removed in vacuo to yield a residue, which was purified by silica gel to afford the pure product as an oil.

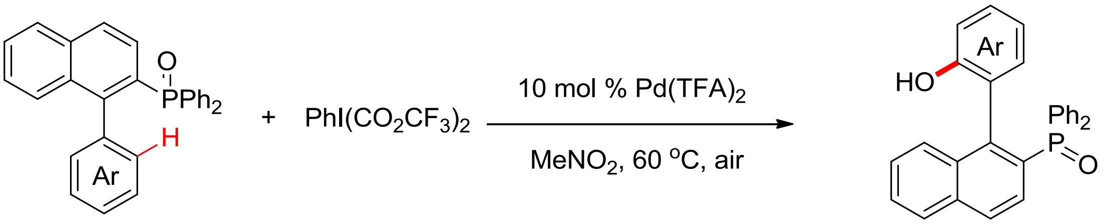

In a similar manner as described before [4]: Under air atmosphere, diarylphosphine oxide (0.30 mmol, 1.0 equiv), $Pd(TFA)_2$ (10.0 mg, 0.03 mmol, 10.0 mol %) and $PhI(OTFA)_2$ (193.5 mg, 0.45 mmol, 1.5 equiv) were added to tube containing a magnetic stir bar. After sealed tube, 2.0 mL CH_3NO_2 was added using a syringe. The mixture was stirred at 60 °C in an oil bath until substrate disappeared as judged by TLC. After cooling to room temperature, the solution was removed in vacuo to yield a residue, which was purified by silica gel using (1:1 EtOAc/hexane) to afford the pure product as oil.

Ⅳ. Characterization of the products

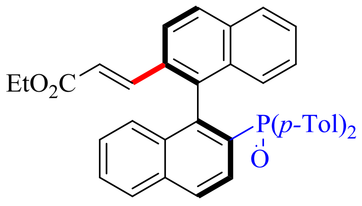

2c

Yellow oil. **^{1}H NMR** (400 MHz, $CDCl_3$) δ: 8.04-8.00 (m, 1 H)， 7.9,5-7.93 (m, 1 H), 7.90-7.85 (m, 1 H), 7.72-7.66 (m, 2 H), 7.59 (d, *J* = 8.8 Hz, 1 H), 7.53-7.50 (m, 1 H), 7.37-7.26 (m, 3 H), 7.23-.08 (m, 5 H), 7.02-7.00 (m, 2 H), 6.95 (t, *J* = 7.2 Hz, 2 H), 6.82 (m, 2 H), 6.19 (d, *J* = 15.9 Hz, 1 H), 4.09 (q, *J* = 7.2 Hz, 2 H), 2.29(s, 3 H), 2.23 (s, 3 H), 1.21 (t, *J* = 7.2 Hz, 3. H); **^{13}C NMR** (100 MHz, $CDCl_3$) δ: 166.51，142.85，141.46，141.44，141.07，141.04，140.93，140.85，137.05，137.01，134.50，134.48，133.68，133.44，133.22，133.10，131.82，131.72，131.50，131.40，131.35，130.50，130.35，129.44，129.32，128.89，128.74，128.62，128.27，128.14，128.08，128.02，127.64，127.42，127.22，126.96，126.58，126.35，122.16，118.37，60.10，21.44，21.36，14.17; **^{31}P NMR** (162 MHz, $CDCl_3$) δ: 28.71; **MS (ESI):** found $[M+Na]^+$603.21 ;$[\alpha]^{22}_D$ = -29.0° (c = 1.0, $CHCl_3$); Enantiomeric excess is 99% determined by HPLC (Chiralcel AD, Hexane/Isopropanol 90/10, flow rate = 1.0 mL/min, 232 nm): major isomer: t_R = 115.72 min， an other isomer was not found.

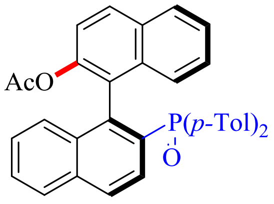

2d

Yellow oil. **^{1}H NMR** (400 MHz, $CDCl_3$) δ: 8.01-7.93 (m, 2 H), 7.90 (d, *J* = 8.2 Hz, 1 H)，7.70 (d, *J* = 9.0 Hz, 1 H), 7.65 (d, *J* = 8.2 Hz, 1 H), 7.52 (t, *J* = 7.3 Hz, 1 H), 7.32-7.19 (m, 7 H), 7.13-7.08 (m, 2 H), 6.94-6.89 (m, 3 H), 6.86-6.83 (m, 2 H), 2.26 (s, 3 H), 2.24 (s, 3 H), 1.79 (s, 3 H); **^{13}C NMR** (100 MHz, $CDCl_3$) δ: 168.83，146.95，141.30，141.27，141.09，141.06，138.33，138.25，134.46，134.44，133.71，132.72，132.60，131.64，131.45，131.02，130.88，130.39，130.01，129.71，129.32，129.04，128.93，128.82，128.64，128.49，128.36，128.19，127.94，127.70，127.50，127.28，126.89，126.37，126.23，125.67，125.63，125.15，121.39，115.63，21.44，21.40，20.75; **^{31}P NMR** (162 MHz, $CDCl_3$) δ: 29.19; **MS (ESI):** found $[M+H]^+$ 541.19;$[\alpha]^{22}_D$ = +58.0° (c = 1.0, $CHCl_3$); Enantiomeric excess is 99% determined by HPLC (Chiralcel AD, Hexane/Isopropanol 85/15, flow rate = 1.0 mL/min, 230.8 nm): major isomer: t_R = 51.69 min, an other isomer was not found.

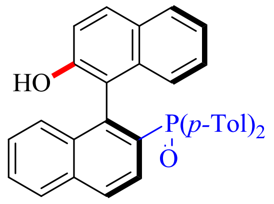

2e

Yellow oil. **^{1}H NMR** (400 MHz, $CDCl_3$) δ: 7.93-7.89 (m, 2 H)，7.674-7.69 (m, 2 H), 7.62 (d, *J* = 8.8 Hz, 1 H), 7.5-7.45 (m, 3 H), 7.30-7.22 (m, 4 H), 7.17-7.02 (m, 4 H), 6.92 (t, *J* = 7.7 Hz, 1 H), 6.54-6.52 (m, 2 H), 6.43 (d, *J* = 8.4 Hz, 1 H), 2.41 (s, 3 H), 2.01 (s, 3 H); **^{13}C NMR** (100 MHz, $CDCl_3$) δ: 153.16，142.83，141.09，141.01，140.92，135.17，133.54，133.39，132.06，131.97，130.74，130.32，129.86，129.75，129.50，129.37，128.88，128.44，128.29，128.23，128.16，128.10，127.95，127.91，127.78，127.45，127.29，126.38，125.63，125.55，125.46，124.36，122.91，121.70，121.08，21.60，21.23; **^{31}P NMR** (162 MHz, $CDCl_3$) δ: 33.26; **MS (ESI):** found $[M+H]^+$ 499.19;$[\alpha]^{22}_{D}$ = +45.0° (c = 1.0, $CHCl_3$); Enantiomeric excess is 99% determined by HPLC (Chiralcel AD, Hexane/Isopropanol 85/15, flow rate = 1.0 mL/min, 236.7 nm): major isomer: t_R = 22.35 min, an other isomer was not found.

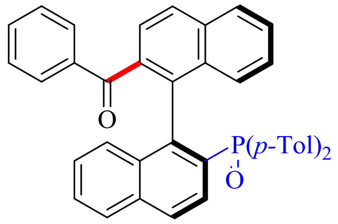

2f

Yellow oil. **^{1}H NMR** (400 MHz, $CDCl_3$) δ: 7.87-7.83 (m, 4 H)，7.72 (d, *J* =7.5 Hz, 2 H), 7.53 (d, *J* =8.6 Hz 1 H), 7.50-7.42 (m, 3 H), 7.36 (t, *J* = 7.4 Hz, 1 H), 7.32-7.24 (m, 4 H), 7.23-7.19 (m, 2 H), 7.16-7.09 (m, 3 H), 7.0-7.01 (m, 3 H), 6.79-6.77 (m, 2 H), 2.28 (s, 3 H), 2.16 (s, 3 H); **^{13}C NMR** (100 MHz, $CDCl_3$) δ: 196.95，143.15，143.08，141.31，141.28，141.23，141.20，137.76，137.48，137.43，135.79，134.54，134.42，133.84，133.71，131.80，131.77，131.70，131.67，131.02，130.68，130.24，129.97，129.18，128.67，128.55，128.41，128.28，127.91，127.84，127.75，127.65，127.63，127.57，127.51，127.30，127.18，127.04，126.86，126.67，126.28，126.18，21.44，21.32; **^{31}P NMR** (162 MHz, $CDCl_3$) δ: 29.05; **MS (ESI):** found $[M+H]^+$ 587.22;$[\alpha]^{22}_{D}$ = +65.0° (c = 1.0, $CHCl_3$); Enantiomeric excess is 99% determined by HPLC (Chiralcel AD, Hexane/Isopropanol 90/10, flow rate = 1.0 mL/min, 233.2 nm): major isomer: t_R = 9.32 min, an other isomer was not found.

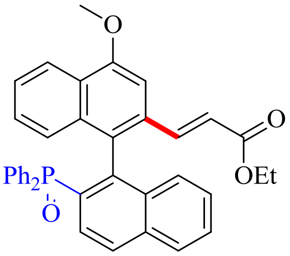

2g

White oil. **^{1}H NMR** (400 MHz, $CDCl_3$) δ: 8.08-8.01 (m, 2 H), 7.94 (d, *J* = 8.2 Hz, 1 H), 7.8,9-7.84 (m, 1 H), 7.54-7.51 (m, 1 H), 7.42-7.38 (m, 2 H), 7.36-7.30 (m, 2 H), 7.27-7.17 (m, 7 H), 7.13-7.01 (m, 4 H), 6.88 (d, *J* = 8.4 Hz, 1 H), 6.82 (s, 1 H), 6.21 (d, *J* =15.8 Hz, 1 H), 4.11 (q, *J* = 7.1 Hz, 2 H), 4.02 (s, 3 H), 1.21 (t, *J* = 7.1 Hz, 3. H); **^{13}C NMR** (100 MHz, $CDCl_3$) δ: 166.47, 155.55, 143.29, 141.24, 141.16, 134.59, 134.57, 134.51, 133.67, 133.65, 133.56, 132.85, 132.62, 131.81, 131.74, 131.64, 131.53, 131.45, 131.35, 131.03, 131.00, 130.62, 130.59, 130.52, 129.97, 129.93, 128.91, 128.80, 128.29, 128.17, 128.10, 128.07, 127.89, 127.77, 127.54, 127.40, 127.30, 127.28, 127.24, 127.17, 126.92, 126.11, 121.69, 118.22, 99.51, 60.14, 55.31, 14.17; **^{31}P NMR** (162 MHz, $CDCl_3$) δ: 28.60; **MS (ESI):** found $[M+H]^+$ 583.20;$[\alpha]^{22}_D$ = -22.0° (c = 0.5, $CHCl_3$); Enantiomeric excess is 98% determined by HPLC (Chiralcel AD, Hexane/Isopropanol 85/15, flow rate = 1.0 mL/min, 209.7 nm): major isomer: t_R = 16.26 min.

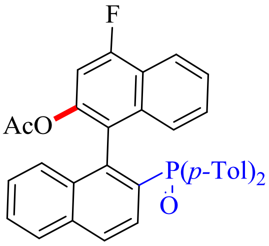

2h

Yellow oil. **^{1}H NMR** (400 MHz, $CDCl_3$) δ: 8.02-7.89 (m, 4 H), 7.55-7.51 (m, 1 H), 7.39-7.35 (m, 1 H), 7.30-7.23 (m, 5 H), 7.18-7.12 (m, 2 H), 6.98-6.89 (m, 6 H), 2.27 (s, 3 H), 1.80 (s, 3 H); **^{13}C NMR** (100 MHz, $CDCl_3$) δ: 168.44, 160.00, 157.48, 146.35, 146.24, 141.41, 141.38, 141.30, 141.27, 137.53, 137.45, 134.52, 134.50, 134.30, 134.25, 132.84, 132.73, 131.77, 131.60, 131.53, 131.49, 131.43, 130.76, 130.49, 129.86, 129.43, 129.05, 128.94, 128.80, 128.49, 128.36, 128.26, 128.22, 128.14, 128.10, 127.82, 127.36, 127.10, 127.02, 126.30, 126.27, 125.46, 121.87, 121.82, 121.48121.32, 120.31, 120.26, 106.32, 106.08, 21.41, 20.70; **^{31}P NMR** (162 MHz, $CDCl_3$) δ: 28.65; **^{19}F NMR** (376 MHz, $CDCl_3$) δ: (d, -120.51); **MS (ESI):** found $[M+H]^+$ 559.20;$[\alpha]^{22}_D$ = -15.0° (c = 0.2, $CHCl_3$); Enantiomeric excess is 99% determined by HPLC (Chiralcel AD, Hexane/Isopropanol 85/15, flow rate = 1.0 mL/min, 233.2 nm): major isomer: t_R = 30.50 min, an other isomer was not found.

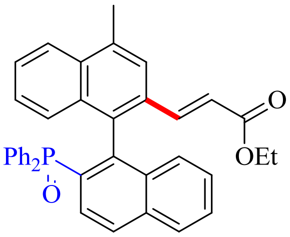

2i

Yellow oil. **^{1}H NMR** (400 MHz, $CDCl_3$) δ: 8.05-8.02 (m, 1 H), 7.94 (d, *J* =8.2 Hz, 1 H), 7.90-7.85(m, 1 H), 7.79 (d, *J* =8.4 Hz, 1 H), 7.53 (t, *J* = 7.8 Hz, 1 H), 7.43-7.32 (m, 5 H), 7.26-7.16 (m, 7 H), 7.12 (t, *J* = 7.8 Hz, 1 H), 7.04-6.97 (m, 4 H), 6.23 (d, *J* =15.9 Hz, 1 H), 4.10 (q, *J* = 7.1 Hz, 2 H), 2.63 (s, 3 H), 1.22 (t, *J* = 7.3 Hz, 3 H); **^{13}C NMR** (100 MHz, $CDCl_3$) δ: 166.55，142.87，141.32，141.23，135.44，135.39，135.14，134.57，134.55，133.75，133.39，133.28，132.86，132.74，132.70，131.84，131.74，131.66，131.45，131.35，131.21，131.13，131.06，131.03，130.38，130.35，130.20，128.91，128.80，128.33，128.21，128.18，128.15，128.09，127.94，127.82，127.37，127.28，127.25，127.17，126.57，126.13，123.80，122.81，118.44，60.10，19.62，14.19; **^{31}P NMR** (162 MHz, $CDCl_3$) δ: 28.71; **MS (ESI):** found $[M+H]^+$ 567.21;$[\alpha]^{22}_{D}$ = -45.0° (c = 0.2, $CHCl_3$); We tried to obtain the enantiomeric excess, but didn't find the corresponding condition.

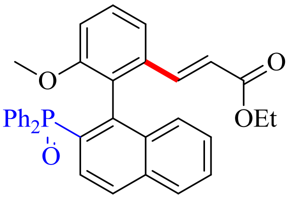

2j

Yellow oil. **^{1}H NMR** (400 MHz, $CDCl_3$) δ:7.87 (d, *J* = 8.3 Hz, 2 H)，7.61-7.50 (m, 6 H), 7.41-.37 (m, 2 H), 7.33-7.17 (m, 8 H), 6.92 (d, *J* = 15.9 Hz, 1 H), 6.71 (d, *J* = 8.1 Hz, 1 H), 6.04 (d, *J* =15.9 Hz, 1 H), 4.03 (q, *J* = 7.2 Hz, 2 H), 3.36 (s, 3 H), 1.16 (t, *J* = 7.2 Hz, 3 H); **^{13}C NMR** (100 MHz, $CDCl_3$) δ: 166.47，155.55，143.29，141.24，141.16，134.59，134.57，134.51，133.67，133.65，133.56，132.85，132.62，131.81，131.74，131.64，131.53，131.45，131.35，131.03，131.00，130.62，130.59，130.52，129.97，129.93，128.91，128.80，128.29，128.17，128.10，128.07，127.89，127.77，127.54，127.40，127.30，127.28，127.24，127.17，126.92，126.11，121.69，118.22，99.51，60.14，55.31，14.17; **^{31}P NMR** (162 MHz, $CDCl_3$) δ: 28.11; **MS (ESI):** found $[M+H]^+$ 533.20;$[\alpha]^{22}_{D}$ = -20.0° (c =0.2, $CHCl_3$); Enantiomeric excess is 74% determined by HPLC (Chiralcel AD-H, Hexane/Isopropanol 90/10, flow rate = 1.0 mL/min, 235.1 nm): major isomer: t_R = 37.82 min, minor isomer: t_R = 41.51 min.

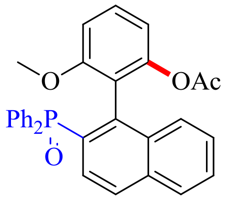

2k

Yellow oil. **^{1}H NMR** (400 MHz, $CDCl_3$) δ: 7.87-7.84 (m, 2 H)，7.68-7.61 (m, 3 H), 7.55-7.50 (m, 3 H), 7.47-7.33 (m, 6 H), 7.31-7.21 (m, 3 H), 6.73 (d, J = 8.2 Hz, 1 H), 6.48 (d, J = 8.3 Hz, 1 H), 3.34 (s, 3 H), 1.62 (s, 3 H); **^{13}C NMR** (100 MHz, $CDCl_3$) δ: 168.53，157.95，149.66，138.14，138.06，134.40，134.38，134.15，133.94，133.12，132.90，132.65，132.54，132.11，132.01，131.87，131.78，131.18，131.15，131.01，130.98，129.57，129.29，128.55，128.43，128.26，127.96，127.92，127.84，127.81，127.69，127.66，127.42，127.30，126.75，126.69，119.11，119.07，114.61，107.15，55.07，20.57; **^{31}P NMR** (162 MHz, $CDCl_3$) δ: 28.39; **^{19}F NMR** (376 MHz, $CDCl_3$) δ: (d, -120.51); **MS (ESI):** found $[M+H]^+$ 493.17;$[\alpha]^{22}_D$ = -10.0° (c = 0.2, $CHCl_3$); Enantiomeric excess is 78% determined by HPLC (Chiralcel AD, Hexane/Isopropanol 85/15, flow rate = 1.0 mL/min, 236.7nm): major isomer: t_R = 24.14 min, minor isomer: t_R = 19.63 min.

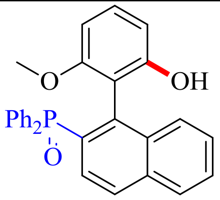

2l

Yellow oil. **^{1}H NMR** (400 MHz, $CDCl_3$) δ: 9.00 (b, 1 H), 7.80-7.68 (m, 4 H), 7.53-7.41 (m, 5 H), 7.38-7.28 (m, 5 H), 7.21-7.16 (m, 2 H), 7.10 (d, J = 2.9 Hz, 1 H), 6.69 (dd, J_1 = 8.8 Hz, J_2 = 3.0 Hz, 1 H), 6.17 (d, J = 8.9 Hz, 1 H), 3.20 (s, 3 H); **^{13}C NMR** (100 MHz, $CDCl_3$) δ: 150.22, 149.46，144.48，144.40, 134.47，133.64, 132.89, 132.77, 132.68，132.61, 132.15, 132.06, 131.63，131.30, 130.79, 130.69，130.47, 129.03, 128,50，128.37, 128.33, 128.21, 127.98, 127.83, 127.78, 127.63, 127.50，127.05，126.92，126.46，125.48，125.42, 121.30, 116.45，109.68，54.40; **^{31}P NMR** (162 MHz, $CDCl_3$) δ: 28.39; **MS (ESI):** found $[M+H]^+$ 451.16;$[\alpha]^{22}_D$ = -20.0° (c = 0.2, $CHCl_3$); Enantiomeric excess is 64% determined by HPLC (Chiralcel OD-H, Hexane/Isopropanol 95/5, flow rate = 0.5 mL/min, 237.9 nm): major isomer: t_R = 19.21 min, minor isomer: t_R = 15.93 min.

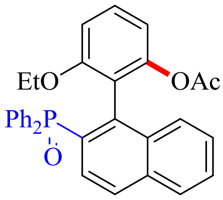

2m

Yellow oil. **^{1}H NMR** (400 MHz, $CDCl_3$) δ: 7.87-7.83 (m, 2 H), 7.66-7.61 (m, 3 H), 7.55-7.45 (m, 4 H), 7.41-7.34 (m, 5 H), 7.31-7.26 (m, 2 H), 7.20 (t, J = 8.2 Hz, 1 H), 6.68 (d, J = 8.2 Hz, 1 H), 6.47 (d, J = 8.4 Hz, 1 H),

3.75-3.59 (m, 2 H), 1.60 (s, 3 H), 0.94 (t, *J* = 6.9 Hz, 3 H); ^{13}**C NMR** (100 MHz, $CDCl_3$) δ: 168.53, 157.37, 149.68，138.62，138.55，134.46，134.44，133.19，133.01，132.66，132.54，132.15，132.05，131.89，131.79，131.49，131.47，131.35，131.32，129.61，128.45，128.34，128.22，128.07，128.05，127.95，127.89，127.77，127.57，127.47，127.34，127.17，126.92，126.63，119.14，119.09，114.41，108.05，63.31，20.45，14.28; ^{31}**P NMR** (162 MHz, $CDCl_3$) δ: 30.53; **MS (ESI):** found $[M+H]^+$ 507.19;$[\alpha]^{22}_D$ = -45.0° (c = 0.2, $CHCl_3$); Enantiomeric excess is 90% determined by HPLC (Chiralcel AD, Hexane/Isopropanol 85/15, flow rate = 1.0 mL/min, 236.7 nm): major isomer: t_R = 22.26 min, minor isomer: t_R = 13.38 min.

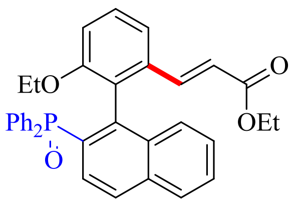

2n

Yellow oil. ^{1}**H NMR** (400 MHz, $CDCl_3$) δ: 7.87 (d, *J* = 8.0 Hz, 2 H)，7.60-7.50 (m, 6 H), 7.43-7.22 (m, 9 H), 7.16 (d, *J* =7.8 Hz, 1 H), 6.92 (d, *J* =15.9 Hz, 1 H), 6.69 (d, *J* =8.1 Hz, 1 H), 6.03 (d, *J* =15.9 Hz, 1 H), 4.03 (q, *J* = 7.0 Hz, 2 H), 3.77-3.64 (m, 2 H), 1.17 (t, *J* = 7.3 Hz, 3 H), 0.92 (t, *J* = 7.2 Hz, 3 H); ^{13}**C NMR** (100 MHz, $CDCl_3$) δ: 166.44，157.16，142.62，140.88，140.81，135.30，134.43，134.41，134.11，133.64，133.10，133.07，132.99，132.62，132.00，131.91，131.86，131.76，131.08，131.05，129.33，128.49，128.37，128.28，127.97，127.93，127.91，127.85，127.79，127.77，127.51，127.47，127.42，127.30，126.80，126.38，118.43，117.41，112.08，63.27，59.90，14.35，14.10; ^{31}**P NMR** (162 MHz, $CDCl_3$) δ: 28.09; **MS (ESI):** found $[M+H]^+$ 547.17;$[\alpha]^{22}_D$ = -25.0° (c = 0.2, $CHCl_3$); Enantiomeric excess is 90% determined by HPLC (Chiralcel IC-3, Agilent 1260, Hexane/Isopropanol 85/15, flow rate = 1.0 mL/min, 240 nm): major isomer: t_R = 36.627 min, minor isomer: t_R = 30.10 min.

Reference

1. Wang, H.-L., Hu, R.-B.; Zhang, H.; Zhou, A.-X.; Yang, S.-D. *Org. Lett.*, **2013**, *15*, 5302-5305, doi: 10.1021/ol402577p.

2. Zhang, H.; Hu, R.-B.; Zhang, X.-Y.; Li, S.-X.; Yang, S.-D. *Chem. Commun.*, **2014**, *50*, 4686-4689, doi;10.1039/C4CC01238K.

3. Ma, Y.-N., Tian, Q.-P., Zhang, H.-Y.; Zhou, A.-X.; Yang, S.-D. *Org. Chem. Front.*, **2014**, *1*, 284-288, doi:10.1039/C4QO00005F.

4. Zhang, H.-Y.; Yi, H.-M.; Wang, G.-W.; Yang, B.; Yang, S.-D. *Org. Lett.*, **2013**, *15*, 6186-6189, doi:10.1021/ol403028a.

Ⅴ. Copies of NMR and HPLC spectra charts

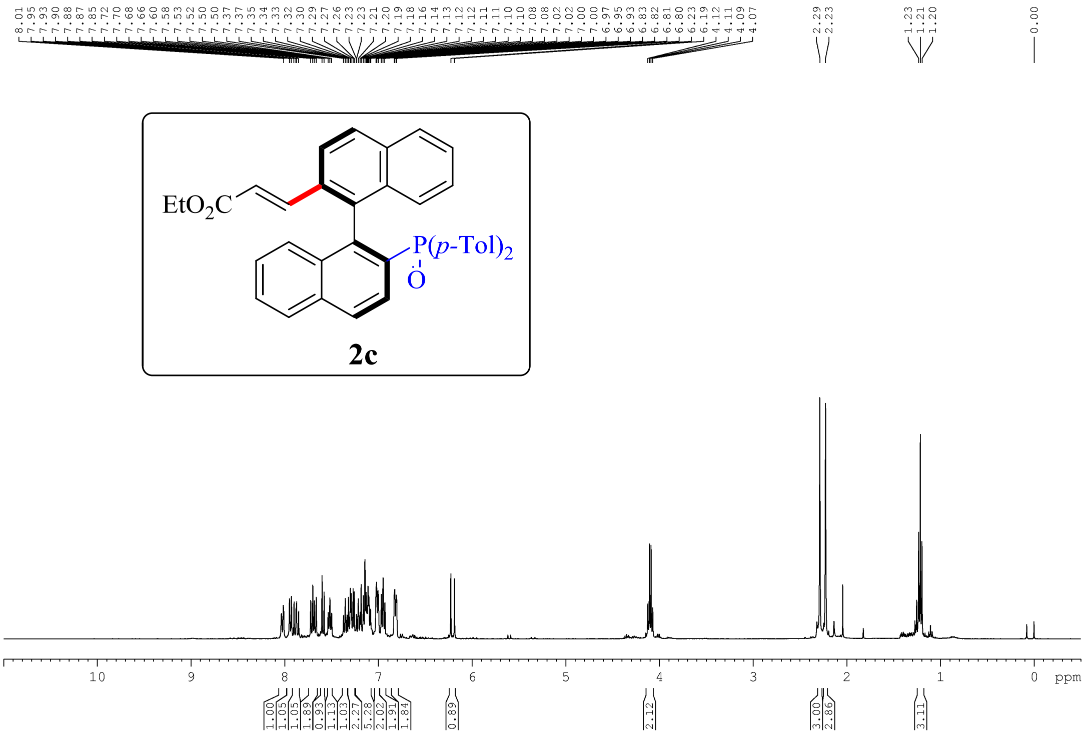

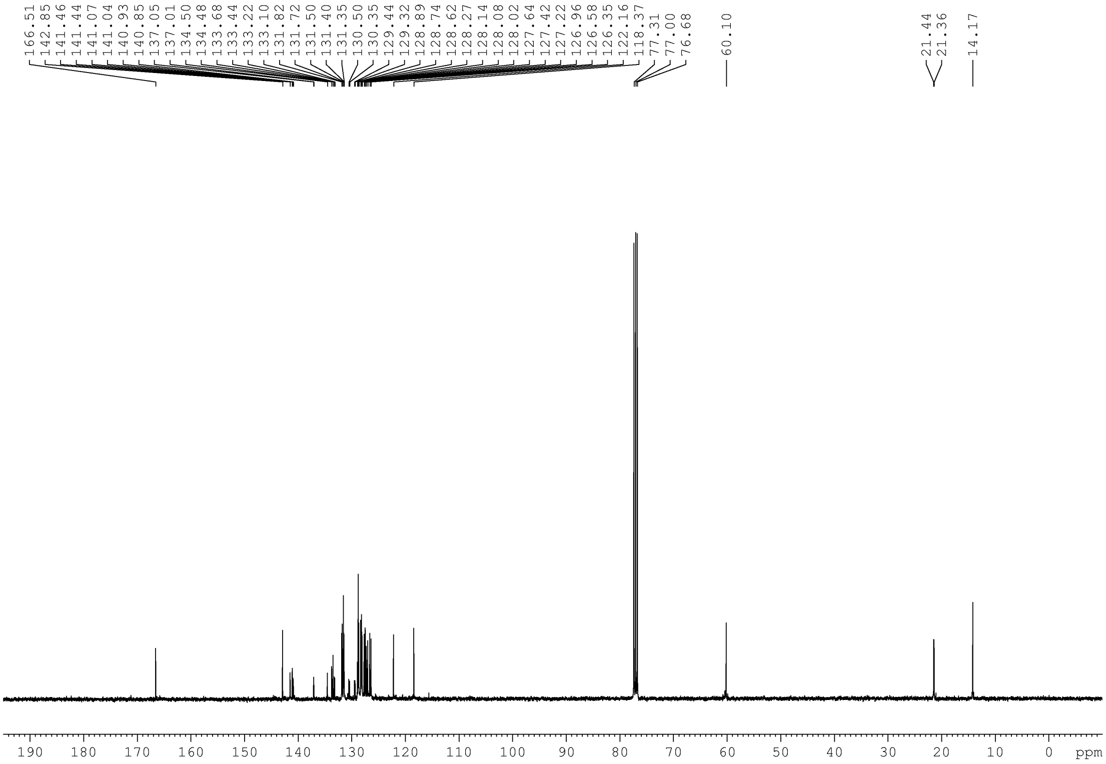

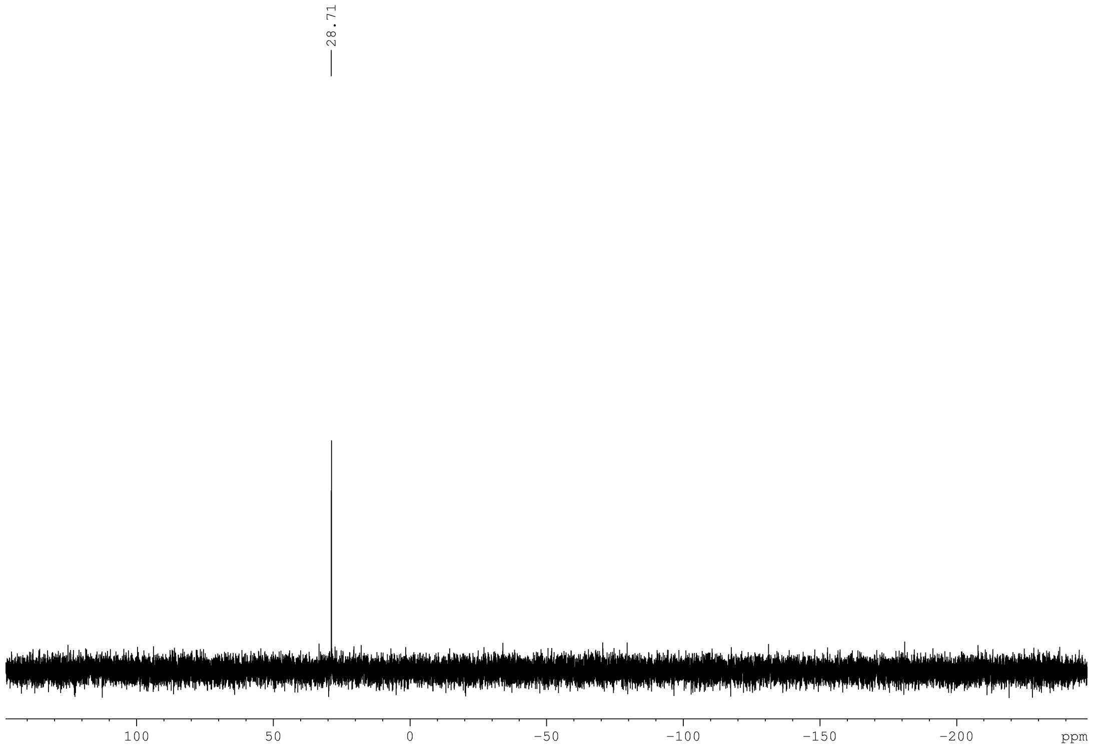

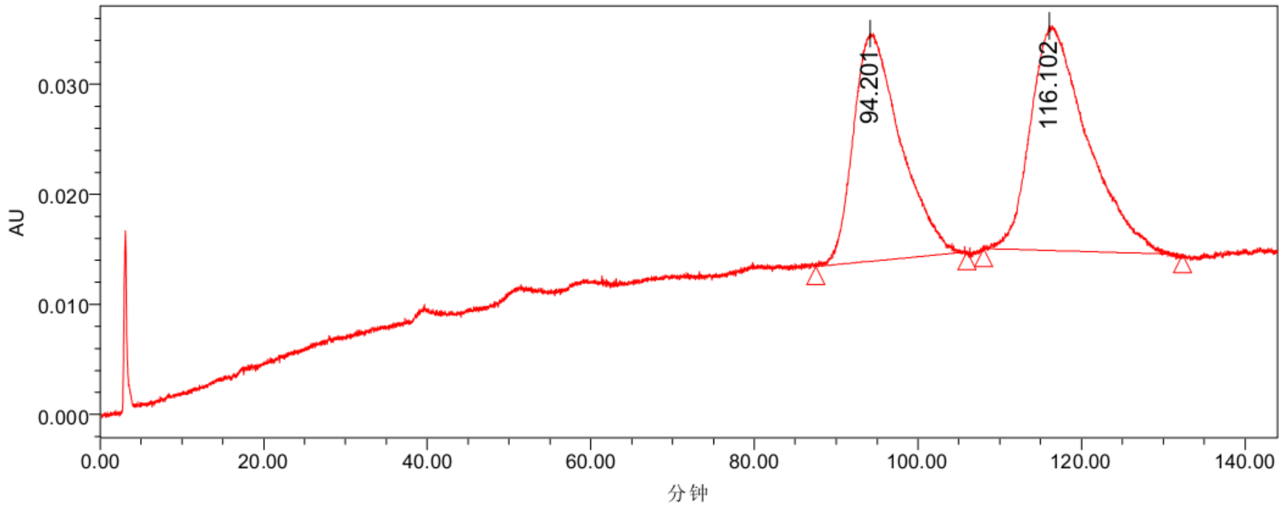

处理通道: PDA 232.0 纳米

	处理通道	保留时间 (分钟)	面积	% 面积	峰高
1	PDA 232.0 纳米	94.201	8307715	45.63	20770
2	PDA 232.0 纳米	116.102	9900071	54.37	20338

Processing channel: PDA 232.0nm

	Processing channel	Retention time (minute)	Area	Area %	Peak height
1	PDA 232.0 nm	94.201	8307715	45.63	20770
2	PDA 232.0 nm	116.102	9900071	54.37	20338

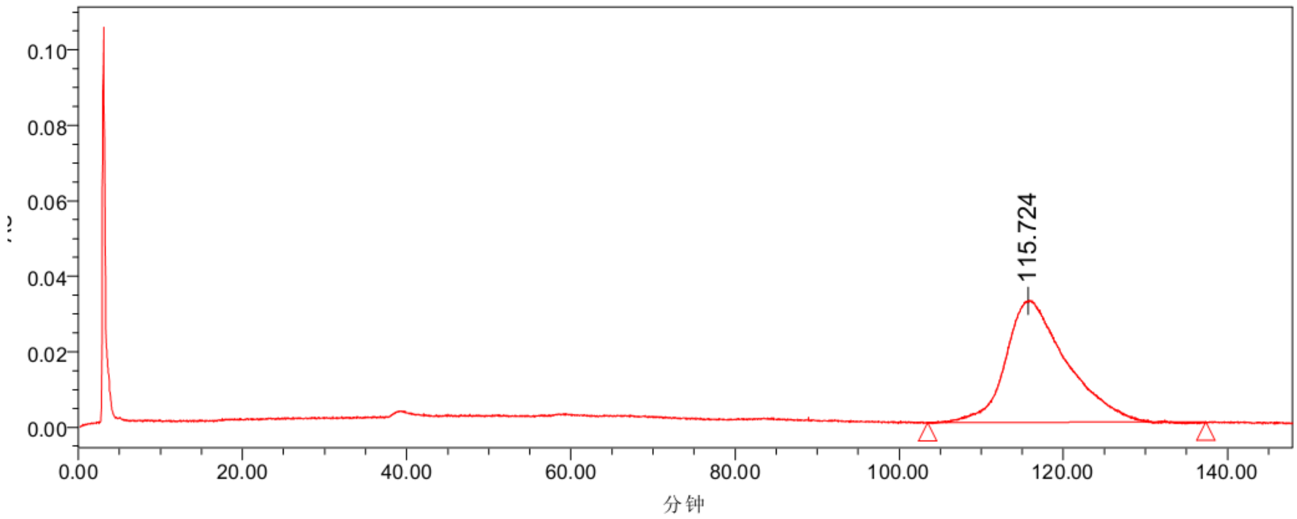

处理通道: PDA 232.0 纳米

	处理通道	保留时间 (分钟)	面积	% 面积	峰高
1	PDA 232.0 纳米	115.724	16693790	100.00	32195

Processing channel: PDA 232.0nm

	Processing channel	Retention time (minute)	Area	Area %	Peak height
1	PDA 232.0nm	115.724	16693790	100.00	32195

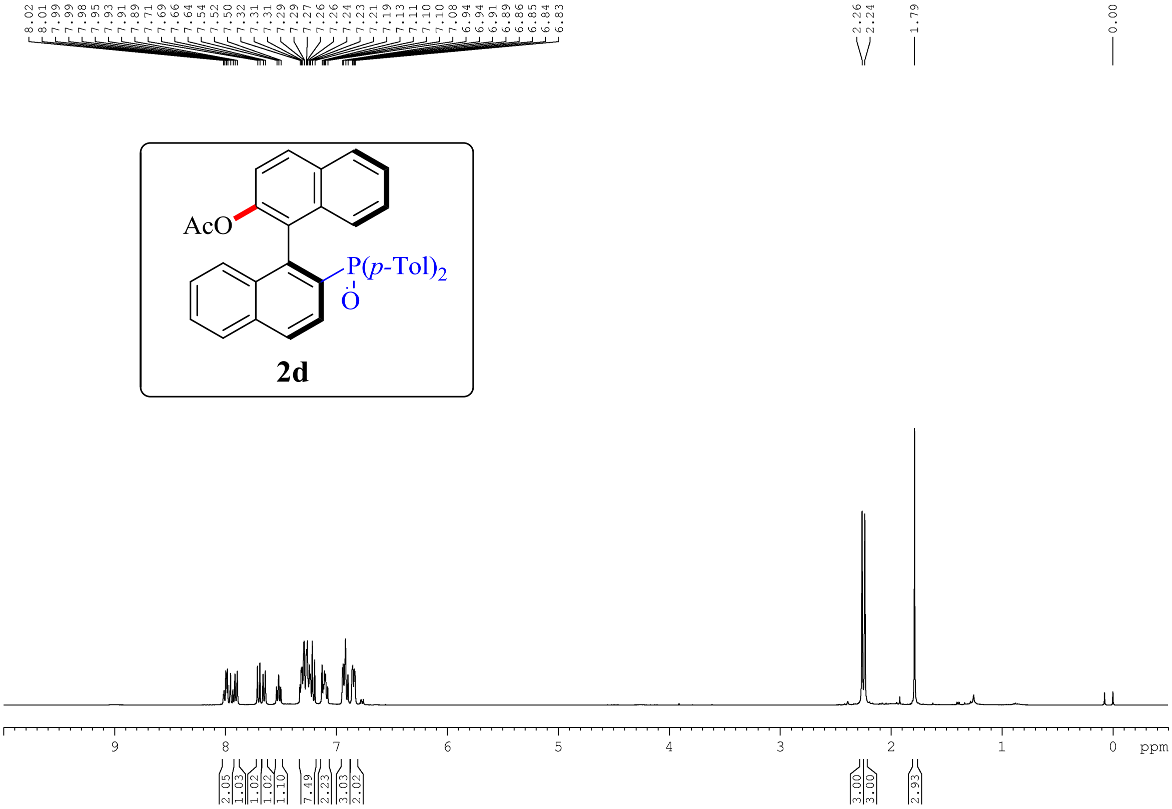
8.02 8.01 7.99 7.99 7.98 7.95 7.93 7.91 7.89 7.71 7.69 7.66 7.64 7.54 7.52 7.50 7.32 7.31 7.31 7.29 7.29 7.27 7.26 7.26 7.24 7.23 7.21 7.19 7.13 7.11 7.10 7.08 6.94 6.94 6.91 6.89 6.86 6.85 6.84 6.83
2.26 2.24
1.79
0.00
AcO
P(p-Tol)2
O
2d
9 8 7 6 5 4 3 2 1 0 ppm
2.05 1.03 1.02 1.02 1.10 7.49 2.23 3.03 2.02
3.00 3.00
2.93

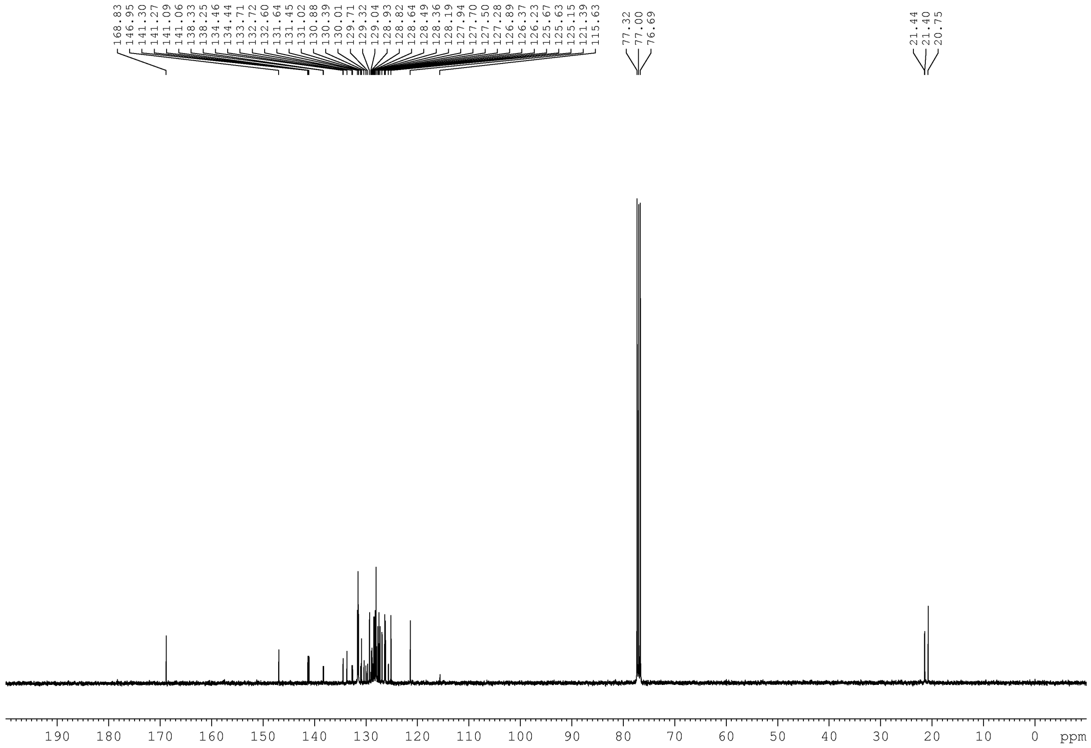
168.83 146.95 141.30 141.27 141.09 141.06 138.33 138.25 134.46 134.44 133.71 132.72 132.60 131.64 131.45 131.02 130.88 130.39 130.01 129.71 129.32 129.04 128.93 128.82 128.64 128.49 128.36 128.19 127.94 127.70 127.50 127.28 126.89 126.37 126.23 125.67 125.63 125.15 121.39 115.63
77.32 77.00 76.69
21.44 21.40 20.75
190 180 170 160 150 140 130 120 110 100 90 80 70 60 50 40 30 20 10 0 ppm

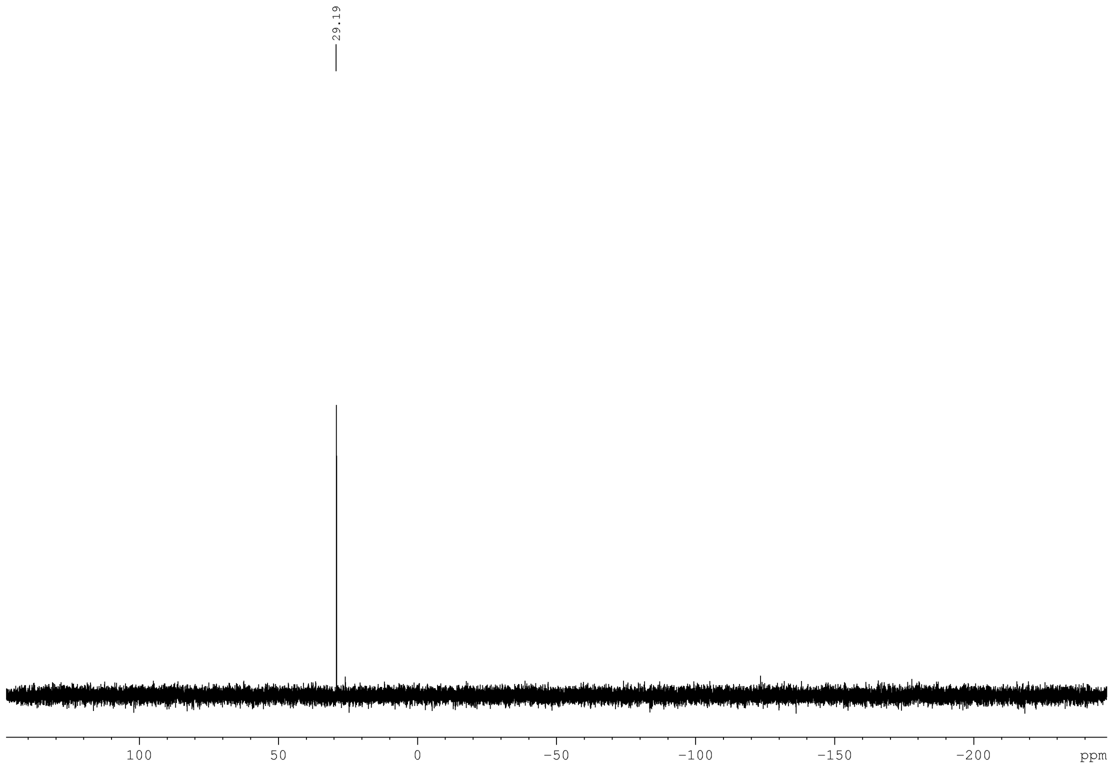

29.19
100
50
0
-50
-100
-150
-200
ppm

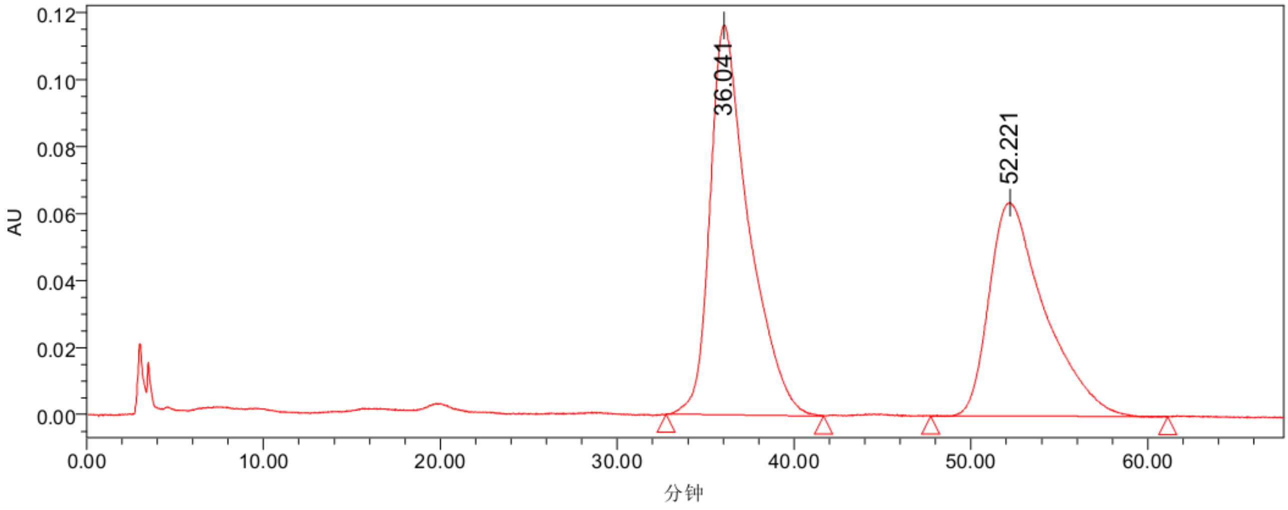

0.12
0.10
0.08
0.06
0.04
0.02
0.00
AU
36.041
52.221
0.00
10.00
20.00
30.00
40.00
50.00
60.00
分钟

处理通道: PDA 230.8 纳米

	处理通道	保留时间 (分钟)	面积	% 面积	峰高
1	PDA 230.8 纳米	36.041	17736728	55.91	116381
2	PDA 230.8 纳米	52.221	13987835	44.09	63774

Processing channel: PDA 230.8nm

	Processing channel	Retention time (minute)	Area	Area %	Peak height
1	PDA 230.8 nm	36.041	17736728	55.91	116381
2	PDA 230.8 nm	52.221	13987835	44.09	63774

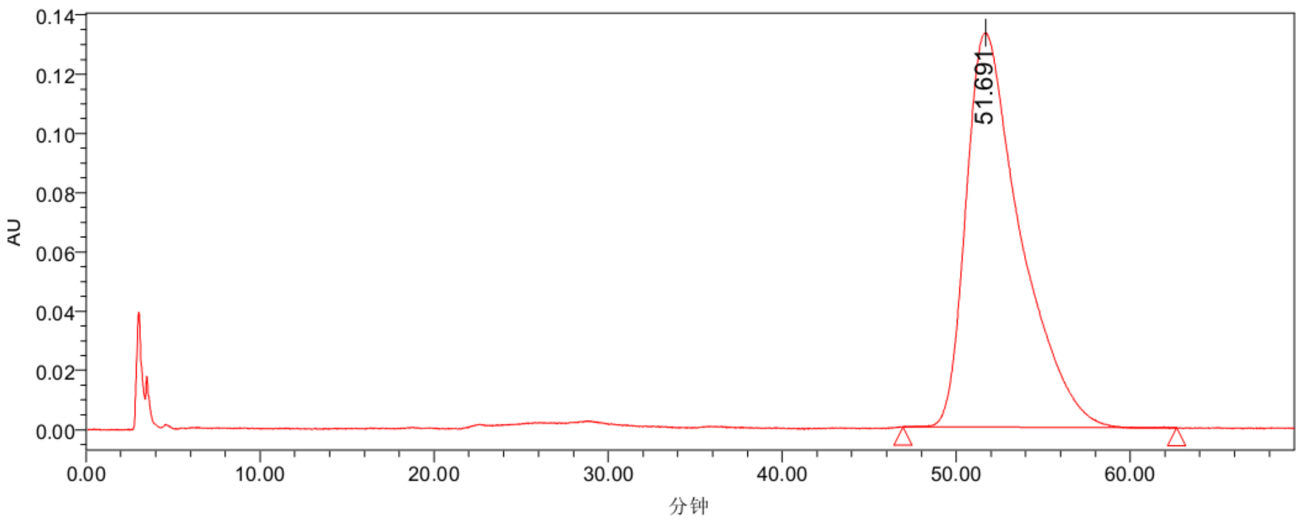

处理通道: PDA 230.8 纳米

	处理通道	保留时间 (分钟)	面积	% 面积	峰高
1	PDA 230.8 纳米	51.691	28979760	100.00	133151

Processing channel: PDA 230.8nm

	Processing channel	Retention time (minute)	Area	Area %	Peak height
1	PDA 230.8nm	51.691	28979760	100.00	133151

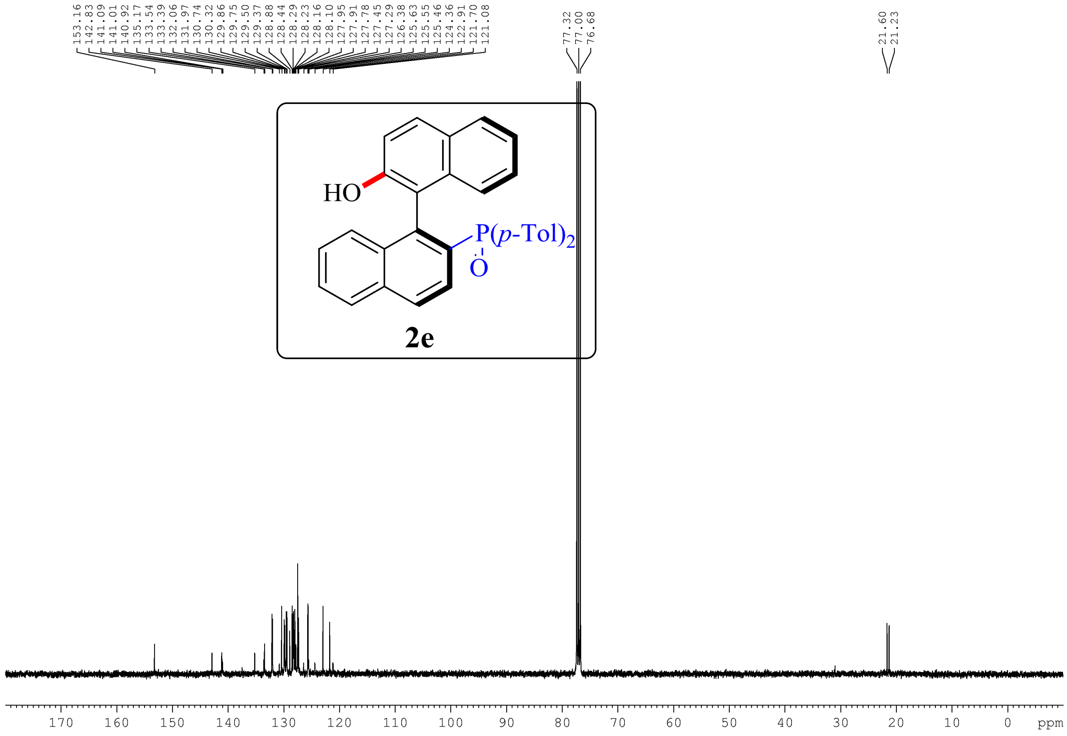
153.16
142.83
141.09
141.01
140.92
135.17
133.54
133.39
132.06
131.97
130.74
130.32
129.86
129.75
129.50
129.37
128.88
128.44
128.29
128.23
128.16
128.10
127.95
127.91
127.78
127.45
127.29
126.38
125.63
125.55
125.46
124.36
122.91
121.70
121.08
77.32
77.00
76.68
21.60
21.23
HO
P(p-Tol)2
O
2e
ppm

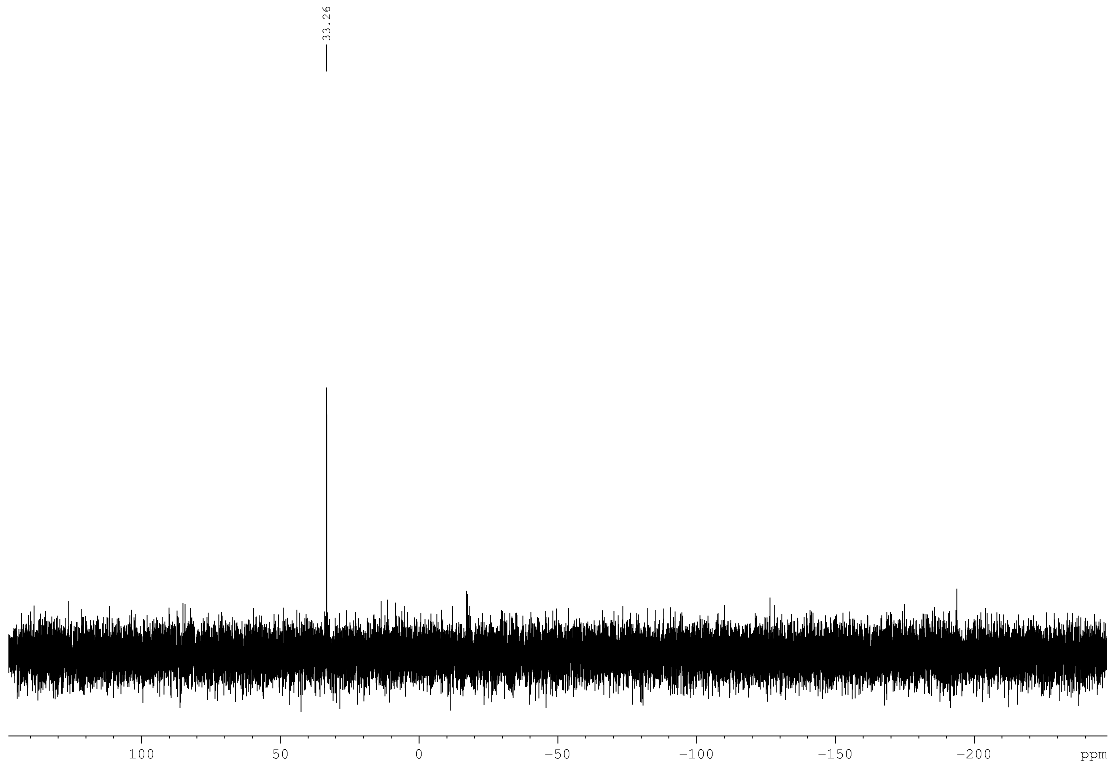
33.26
ppm

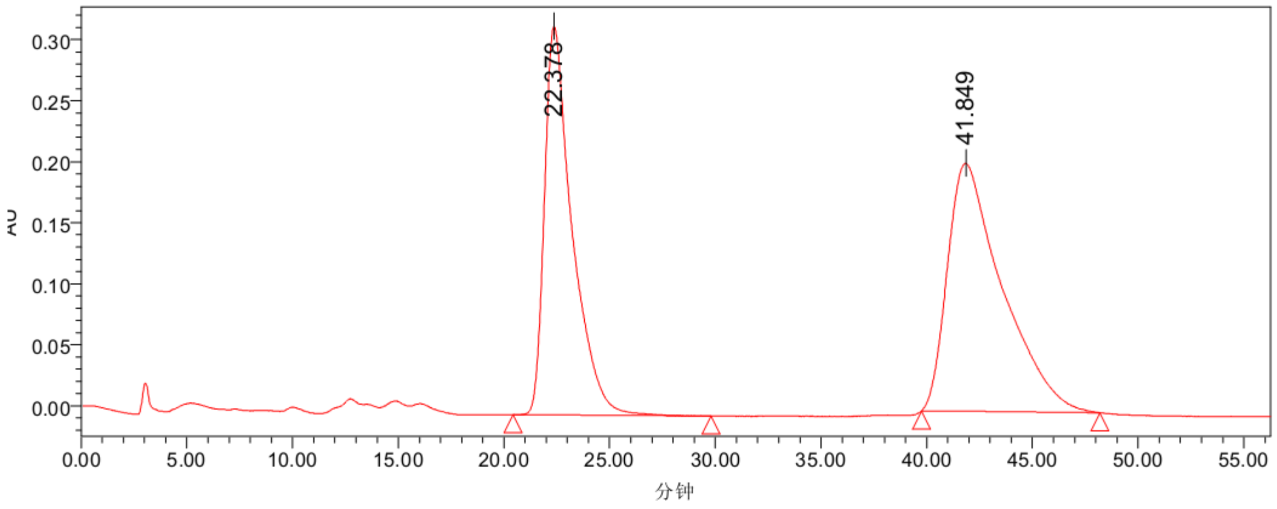

处理通道: PDA 236.7 纳米

	处理通道	保留时间 (分钟)	面积	% 面积	峰高
1	PDA 236.7 纳米	22.378	29034173	44.20	318496
2	PDA 236.7 纳米	41.849	36661096	55.80	203379

Processing channel: PDA 236.7nm

	Processing channel	Retention time (minute)	Area	Area %	Peak height
1	PDA 236.7nm	22.378	29034173	44.20	318496
2	PDA 236.7 nm	41.849	36661096	55.80	203379

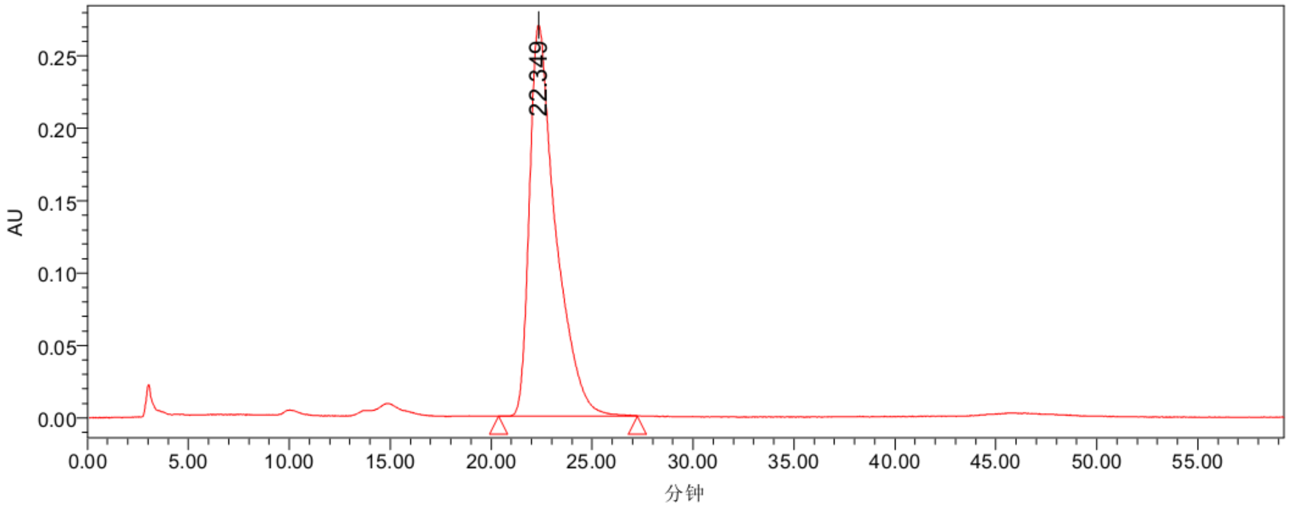

处理通道: PDA 236.7 纳米

	处理通道	保留时间 (分钟)	面积	% 面积	峰高
1	PDA 236.7 纳米	22.349	24311437	100.00	270218

Processing channel: PDA 236.7nm

	Processing channel	Retention time (minute)	Area	Area %	Peak height
1	PDA 236.7nm	22.349	24311437	100.00	270218

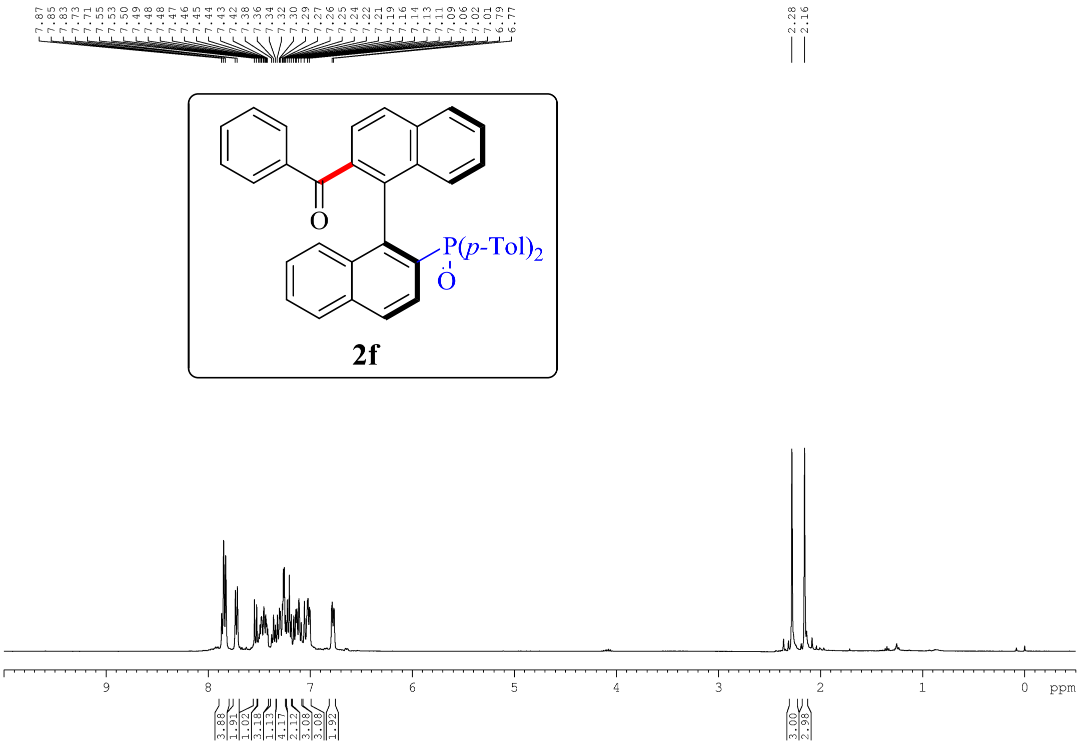

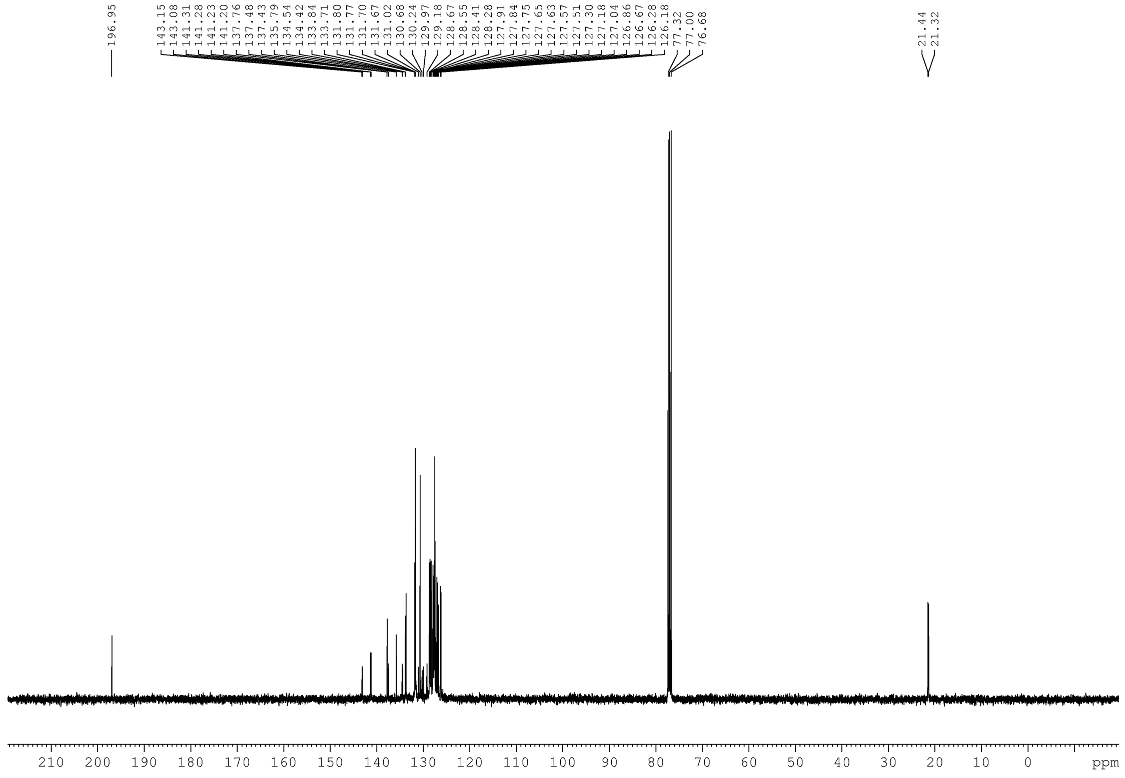
196.95
143.15
143.08
141.31
141.28
141.23
141.20
137.76
137.48
137.43
135.79
134.54
134.42
133.84
133.71
131.80
131.77
131.70
131.67
131.02
130.68
130.24
129.97
129.18
128.67
128.55
128.41
128.28
127.91
127.84
127.75
127.65
127.63
127.57
127.51
127.30
127.18
127.04
126.86
126.67
126.28
126.18
77.32
77.00
76.68
21.44
21.32
ppm

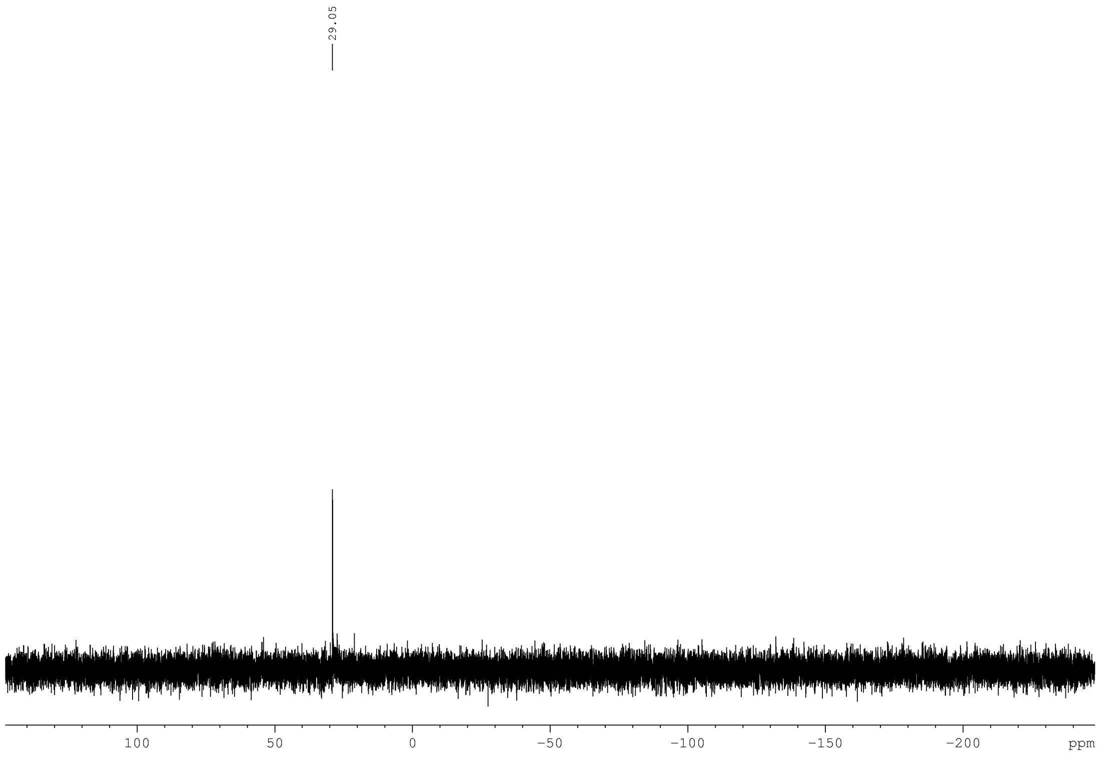
29.05
ppm

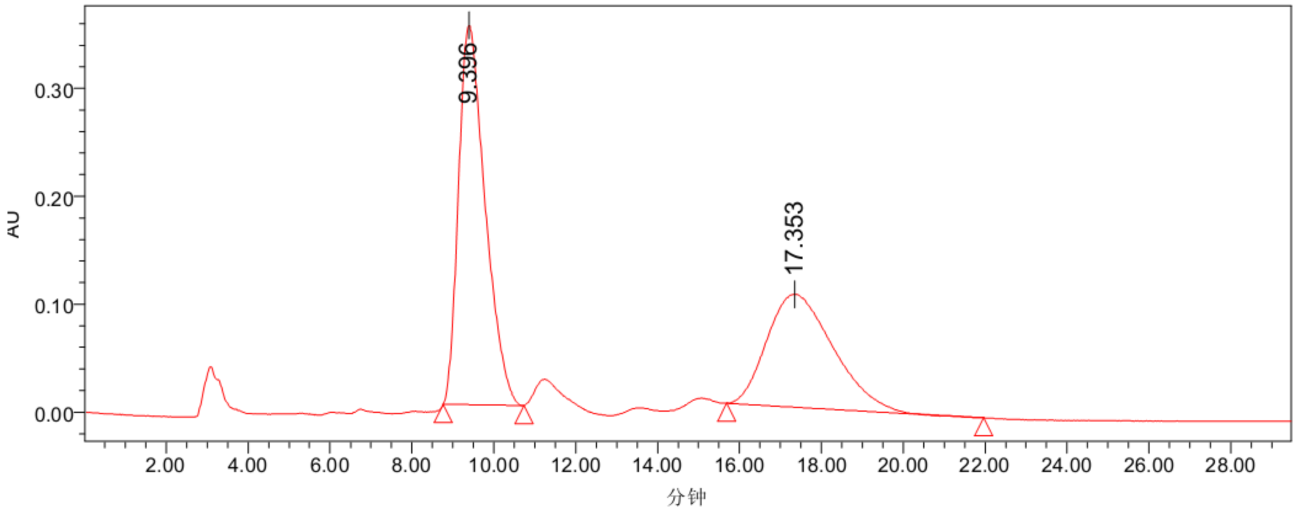

处理通道: PDA 233.2 纳米

	处理通道	保留时间 (分钟)	面积	% 面积	峰高
1	PDA 233.2 纳米	9.396	15900546	57.35	351301
2	PDA 233.2 纳米	17.353	11822919	42.65	104642

Processing channel: PDA 233.2nm

	Processing channel	Retention time (minute)	Area	Area %	Peak height
1	PDA 233.2nm	9.396	29034173	57.35	351301
2	PDA 233.2nm	17.353	11822919	42.65	104642

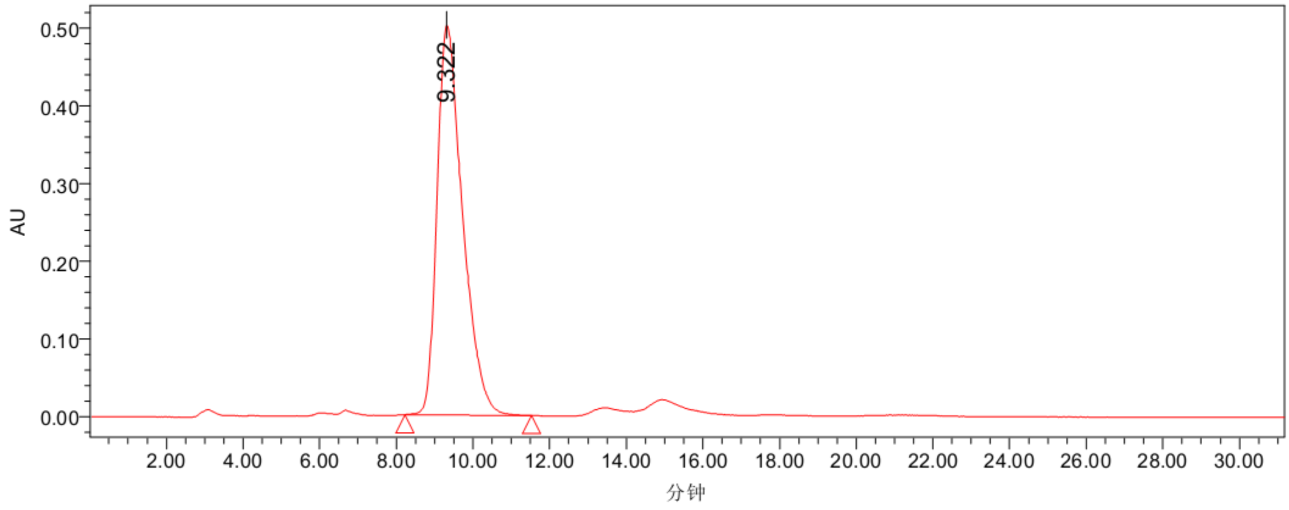

处理通道: **PDA 233.2 纳米**

	处理通道	保留时间 (分钟)	面积	% 面积	峰高
1	PDA 233.2 纳米	9.322	23204244	100.00	502337

Processing channel: PDA 233.2nm

	Processing channel	Retention time (minute)	Area	Area %	Peak height
1	PDA 233.2nm	9.322	23204244	100.00	502337

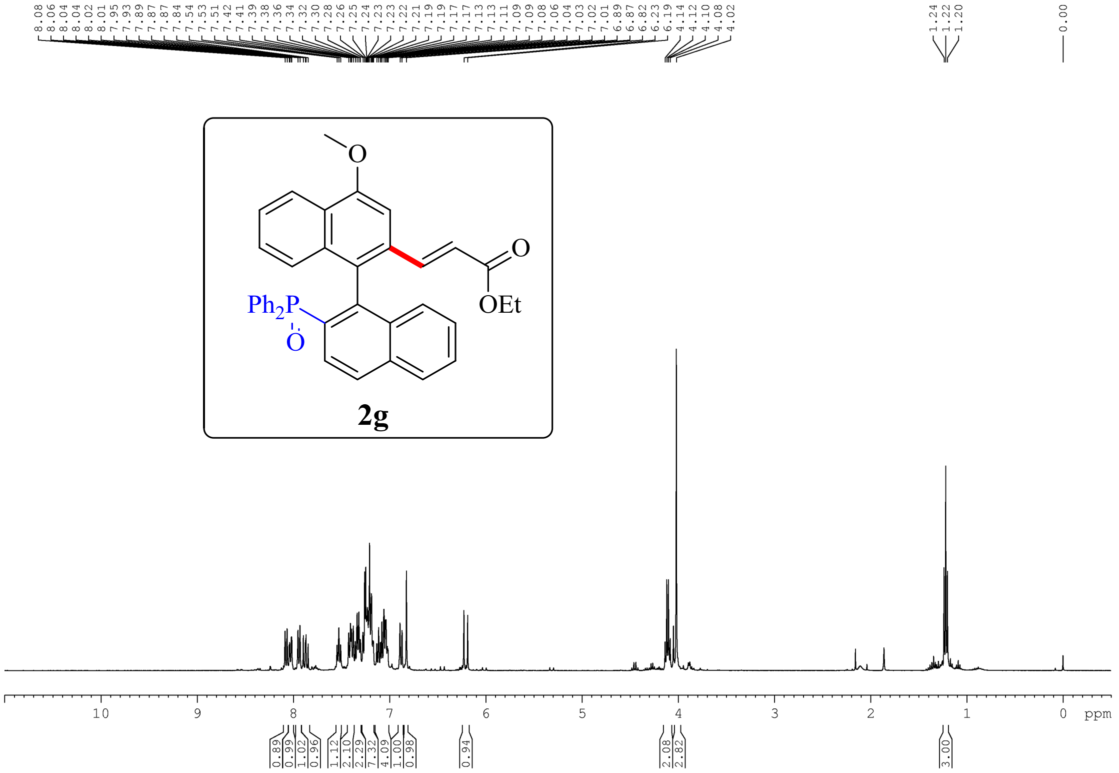
8.08
8.06
8.04
8.04
8.02
8.01
7.95
7.93
7.89
7.87
7.87
7.84
7.54
7.53
7.51
7.42
7.41
7.39
7.38
7.36
7.34
7.32
7.30
7.28
7.26
7.25
7.24
7.23
7.23
7.22
7.21
7.19
7.19
7.17
7.17
7.13
7.13
7.11
7.09
7.09
7.08
7.06
7.04
7.03
7.02
7.01
6.89
6.87
6.82
6.23
6.19
4.14
4.12
4.10
4.08
4.02
1.24
1.22
1.20
0.00
O
O
OEt
Ph2P
O
2g
10
9
8
7
6
5
4
3
2
1
0
ppm
0.89
0.99
1.02
0.96
1.12
2.10
2.29
7.32
4.09
1.00
0.98
0.94
2.08
2.82
3.00

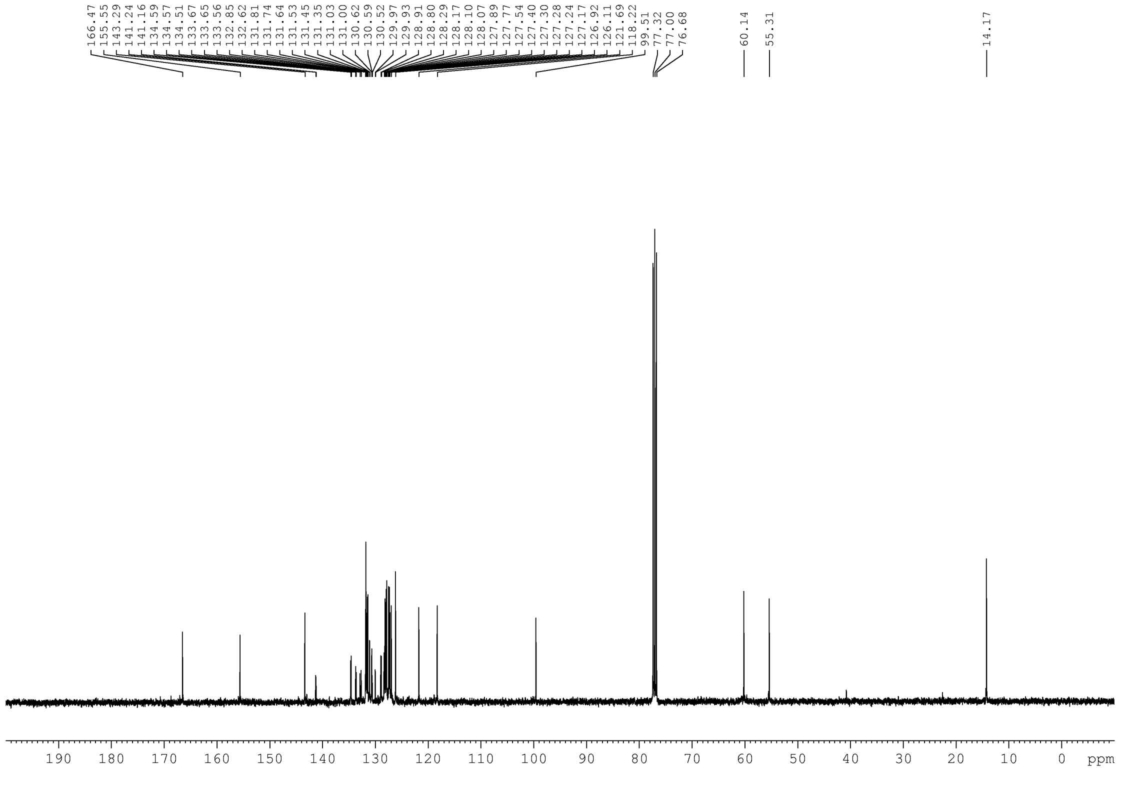
166.47
155.55
143.29
141.24
141.16
134.59
134.57
134.51
133.67
133.65
133.56
132.85
132.62
131.81
131.74
131.64
131.53
131.45
131.35
131.03
131.00
130.62
130.59
130.52
129.97
129.93
128.91
128.80
128.29
128.17
128.10
128.07
127.89
127.77
127.54
127.40
127.30
127.28
127.24
127.17
126.92
126.11
121.69
118.22
99.51
77.32
77.00
76.68
60.14
55.31
14.17
190
180
170
160
150
140
130
120
110
100
90
80
70
60
50
40
30
20
10
0
ppm

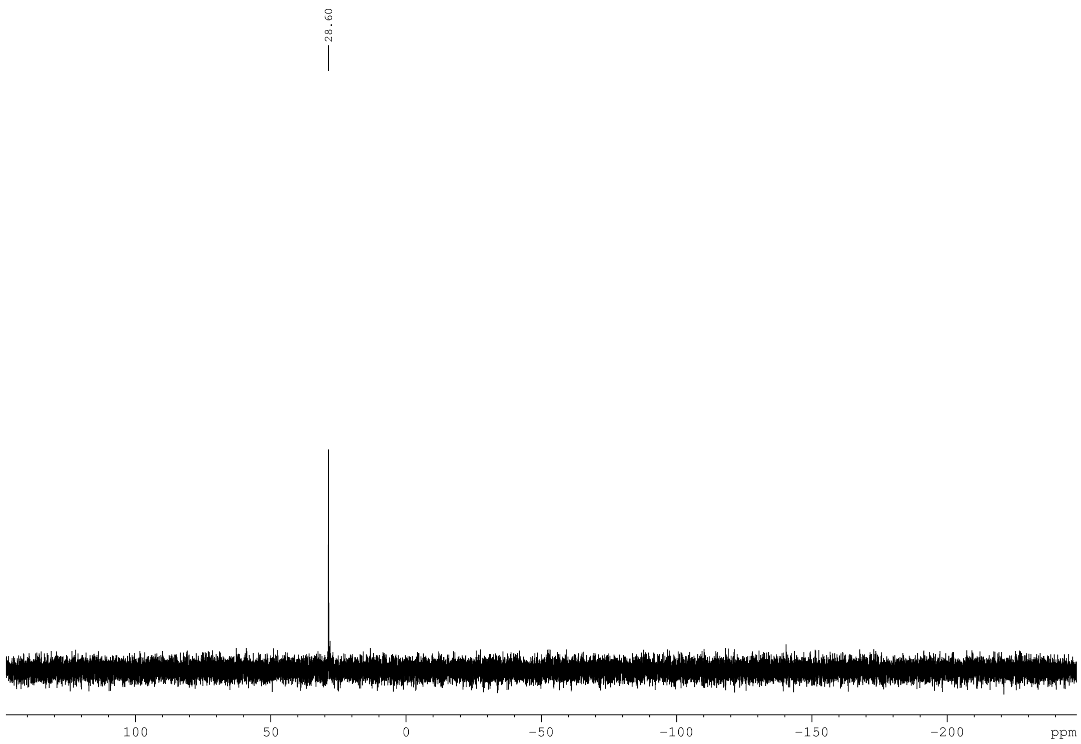

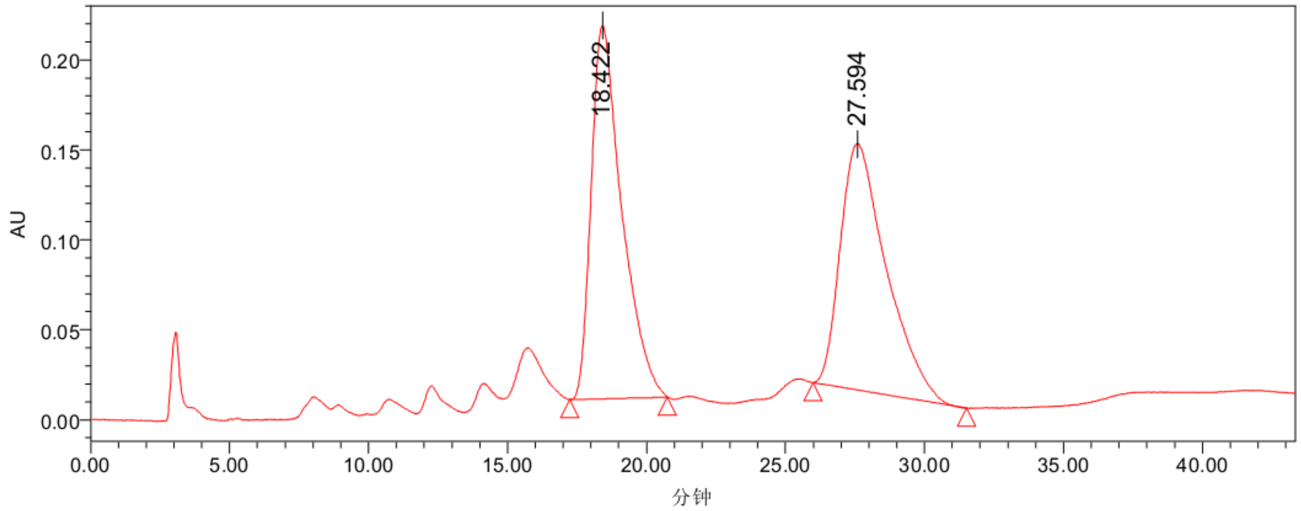

处理通道: PDA 237.9 纳米

	处理通道	保留时间 (分钟)	面积	% 面积	峰高
1	PDA 237.9 纳米	18.422	15886727	50.18	207417
2	PDA 237.9 纳米	27.594	15774759	49.82	136827

Processing channel: PDA 237.9nm

	Processing channel	Retention time (minute)	Area	Area %	Peak height
1	PDA 237.9nm	18.422	15886727	50.18	207417
2	PDA 237.9nm	27.594	15774759	49.82	136827

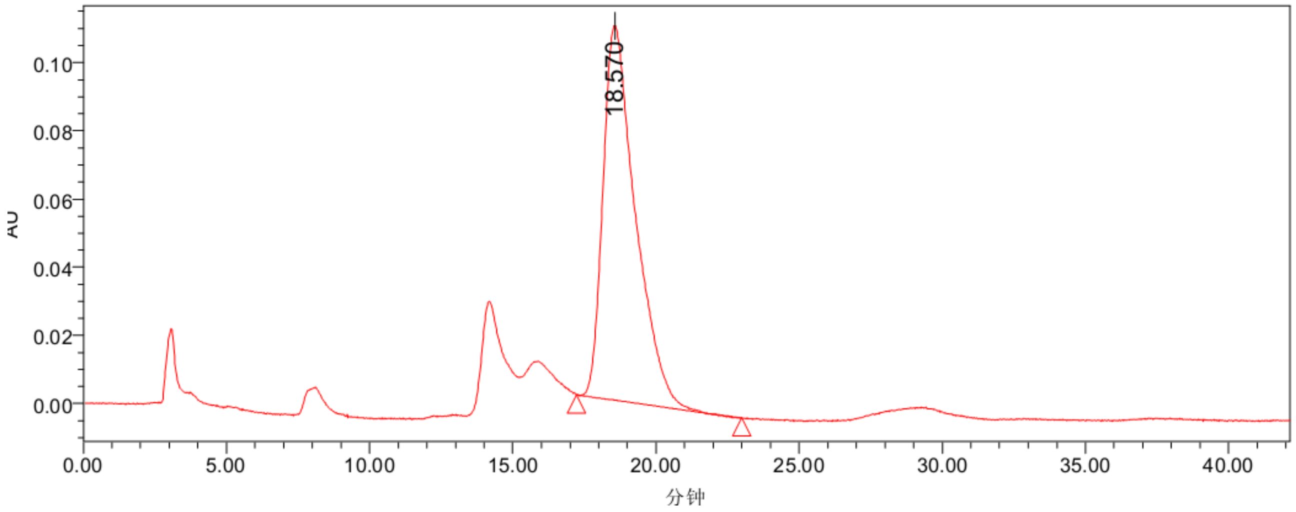

处理通道: PDA 237.9 纳米

	处理通道	保留时间 (分钟)	面积	% 面积	峰高
1	PDA 237.9 纳米	18.570	8670714	100.00	109947

Processing channel: PDA 237.9nm

	Processing channel	Retention time (minute)	Area	Area %	Peak height
1	PDA 237.9nm	18.570	8670714	100.00	109947

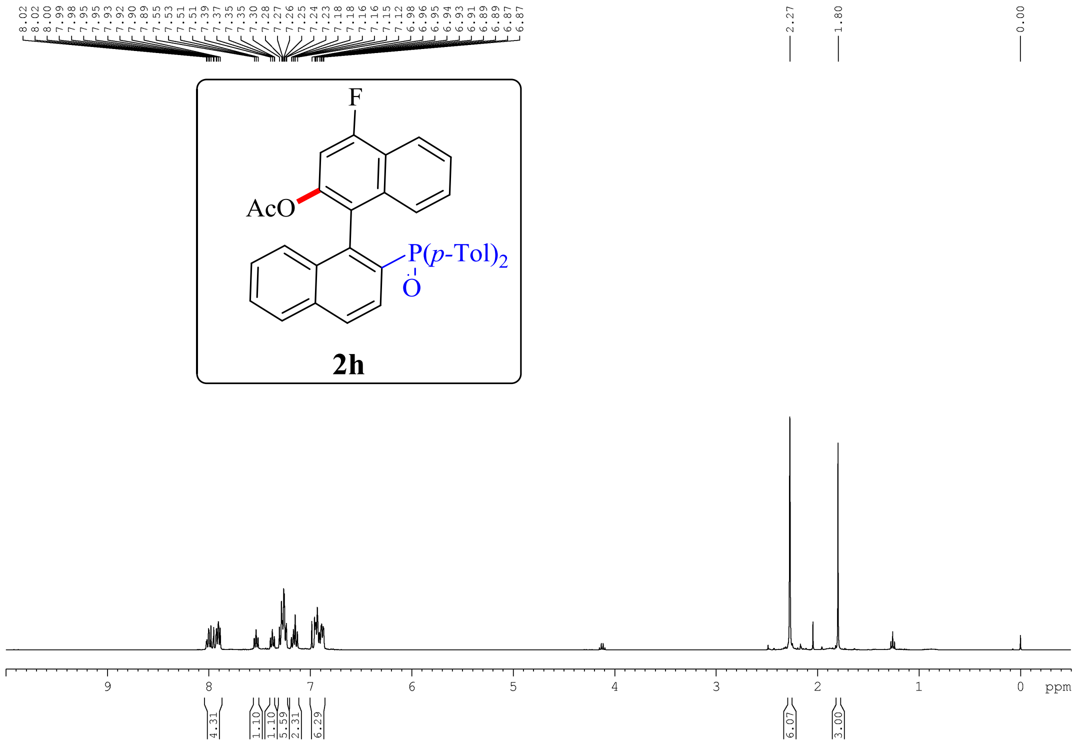
8.02 8.02 8.00 7.99 7.98 7.95 7.95 7.93 7.92 7.90 7.89 7.55 7.53 7.51 7.51 7.39 7.37 7.35 7.35 7.30 7.28 7.27 7.26 7.25 7.24 7.23 7.18 7.16 7.16 7.15 7.12 6.98 6.96 6.95 6.94 6.93 6.91 6.89 6.89 6.87 6.87
2.27
1.80
0.00
F
AcO
P(p-Tol)2
O
2h
9 8 7 6 5 4 3 2 1 0 ppm
4.31 1.10 1.10 5.59 2.31 6.29 6.07 3.00

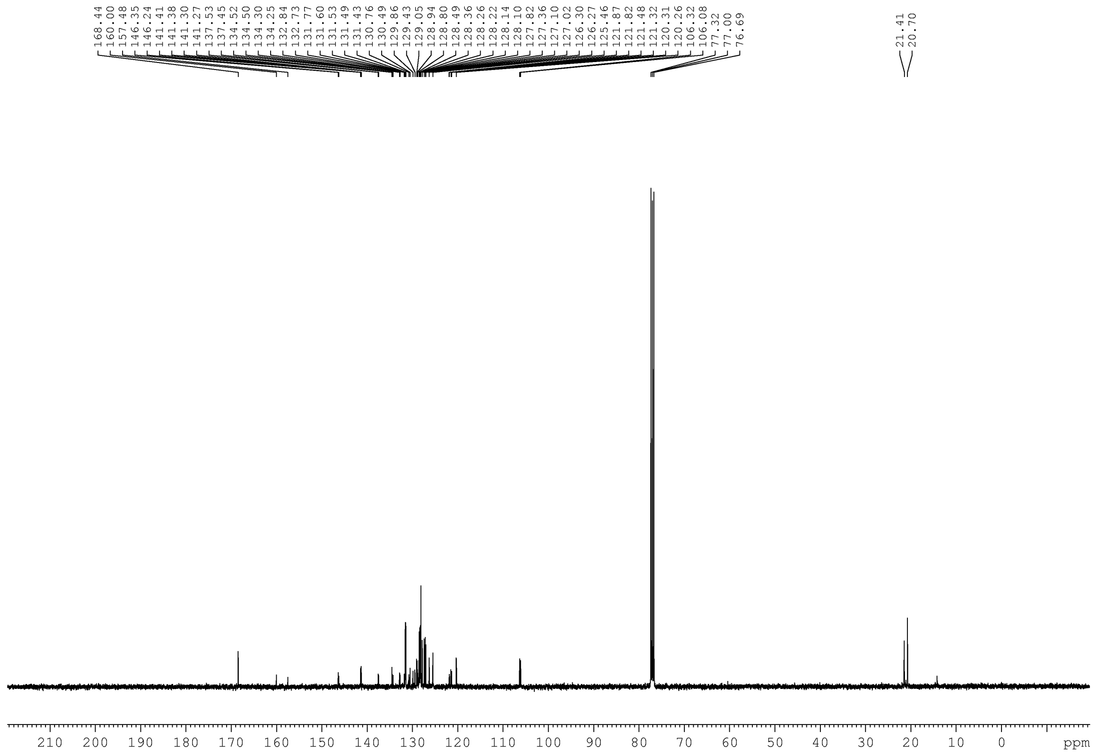
168.44 160.00 157.48 146.35 146.24 141.41 141.38 141.30 141.27 137.53 137.45 134.52 134.50 134.30 134.25 132.84 132.73 131.77 131.60 131.53 131.49 131.43 130.76 130.49 129.86 129.43 129.05 128.94 128.80 128.49 128.36 128.26 128.22 128.14 128.10 127.82 127.36 127.10 127.02 126.30 126.27 125.46 121.87 121.82 121.48 121.32 120.31 120.26 106.32 106.08 77.32 77.00 76.69
21.41 20.70
210 200 190 180 170 160 150 140 130 120 110 100 90 80 70 60 50 40 30 20 10 0 ppm

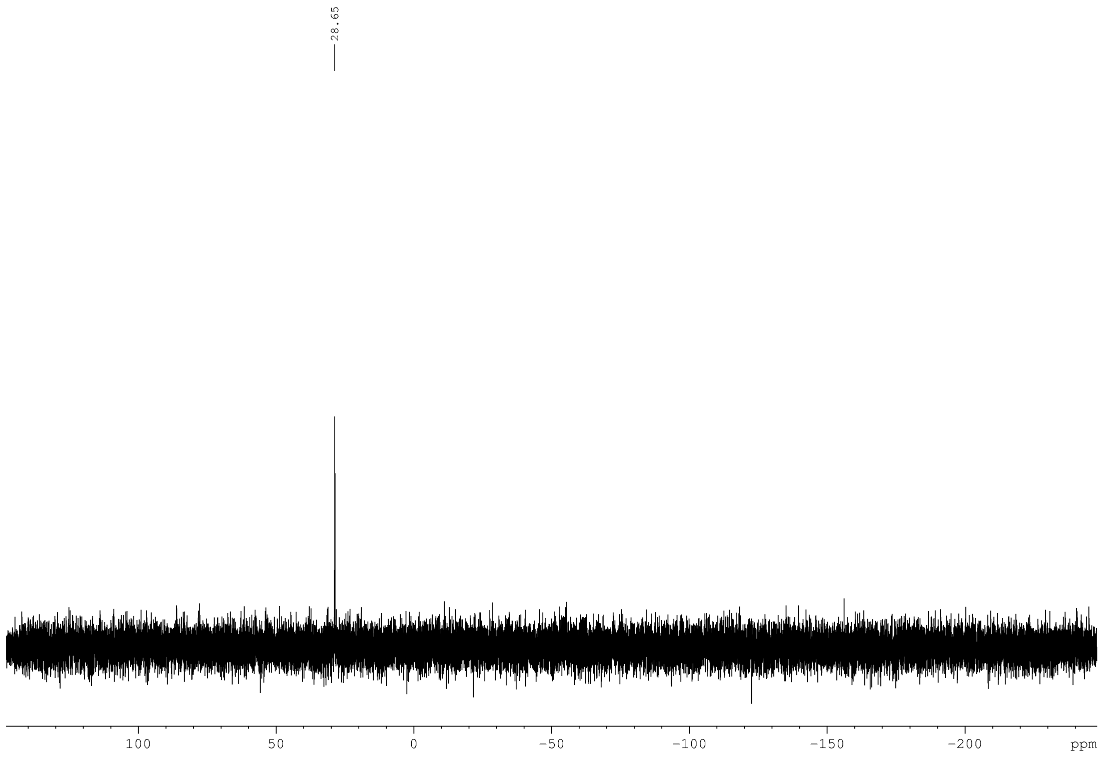
28.65
100
50
0
−50
−100
−150
−200
ppm

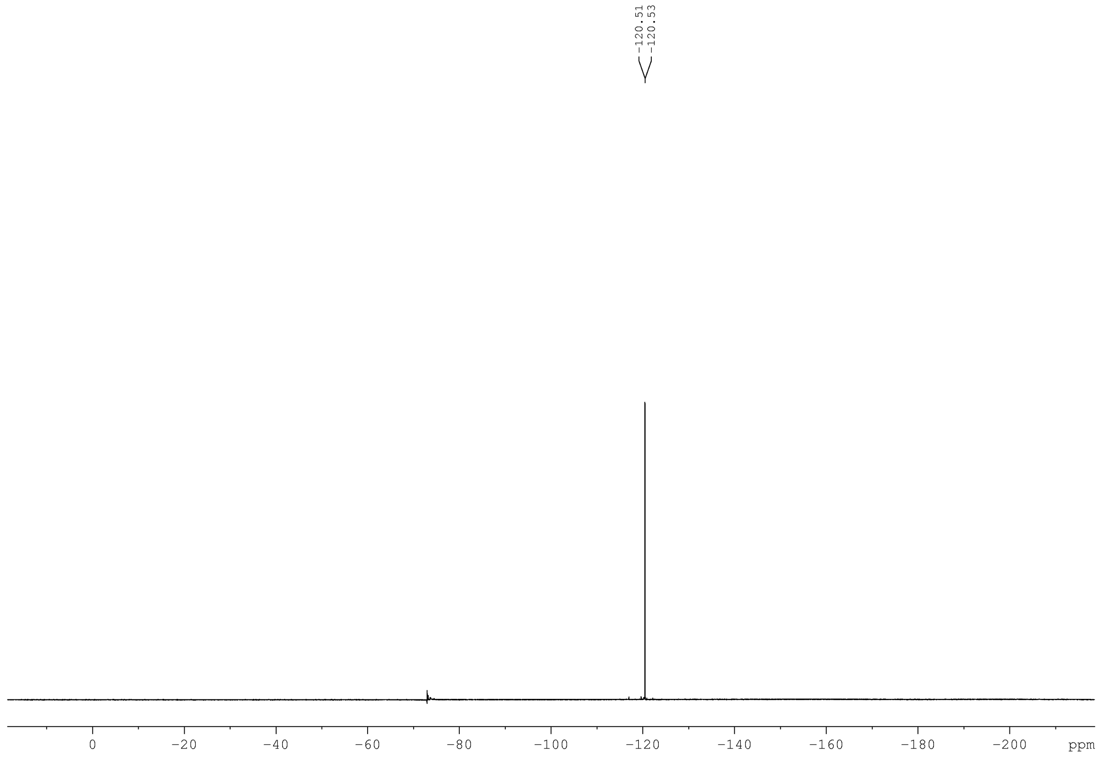
−120.51
−120.53
0
−20
−40
−60
−80
−100
−120
−140
−160
−180
−200
ppm

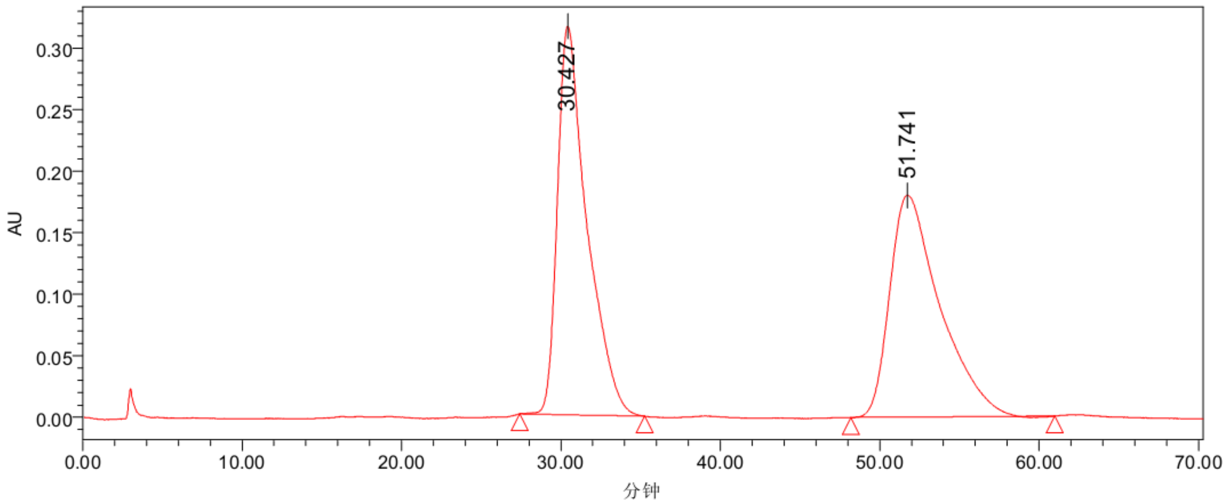

处理通道: **PDA 233.2** 纳米

	处理通道	保留时间 (分钟)	面积	% 面积	峰高
1	PDA 233.2 纳米	30.427	40709312	50.72	315869
2	PDA 233.2 纳米	51.741	39558129	49.28	180431

Processing channel: PDA 233.2nm

	Processing channel	Retention time (minute)	Area	Area %	Peak height
1	PDA 233.2nm	30.427	40709312	50.72	315869
2	PDA 233.2nm	51.741	39558129	48.28	180431

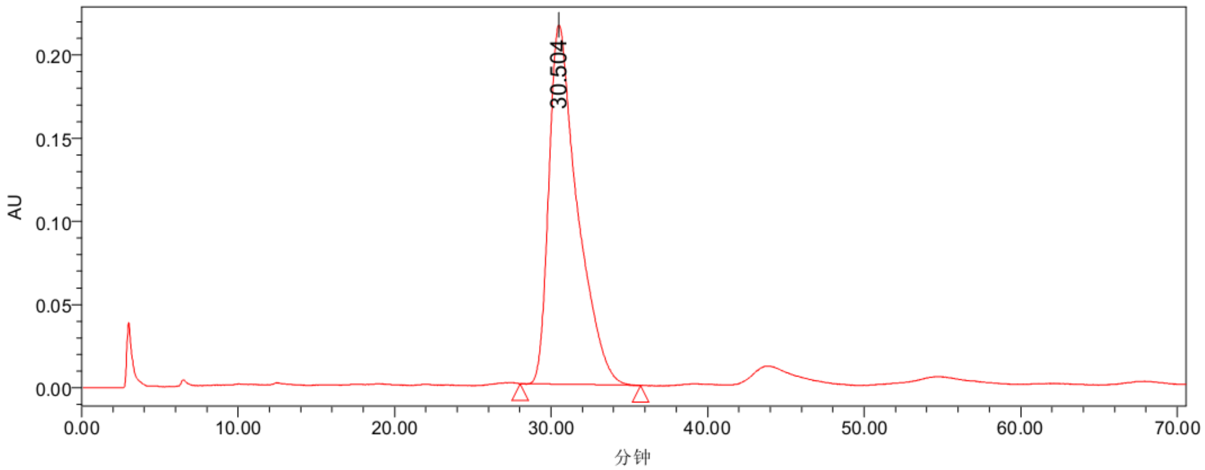

处理通道: PDA 233.2 纳米

	处理通道	保留时间 (分钟)	面积	% 面积	峰高
1	PDA 233.2 纳米	30.504	27347121	100.00	216054

Processing channel: PDA 233.2nm

	Processing channel	Retention time (minute)	Area	Area %	Peak height
1	PDA 233.2nm	30.504	27347121	100.00	216054

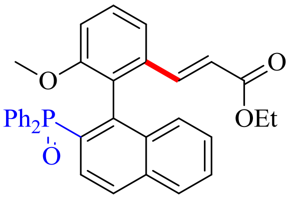
Ph2P
O
O
O
OEt

2j

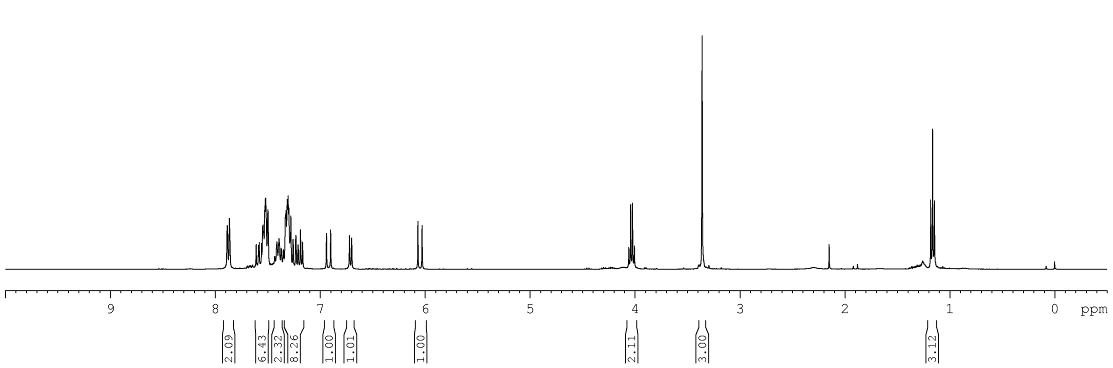
7.89 7.86 7.61 7.59 7.58 7.56 7.54 7.54 7.53 7.53 7.52 7.52 7.50 7.50 7.41 7.40 7.39 7.37 7.37 7.35 7.33 7.32 7.31 7.31 7.30 7.29 7.28 7.26 7.23 7.21 7.19 7.17 6.94 6.90 6.72 6.70 6.07 6.03
4.06 4.04 4.02 4.00
3.36
1.18 1.16 1.15
0.00
9 8 7 6 5 4 3 2 1 0 ppm
2.09 6.43 2.32 8.26 1.00 1.01 1.00 2.11 3.00 3.12

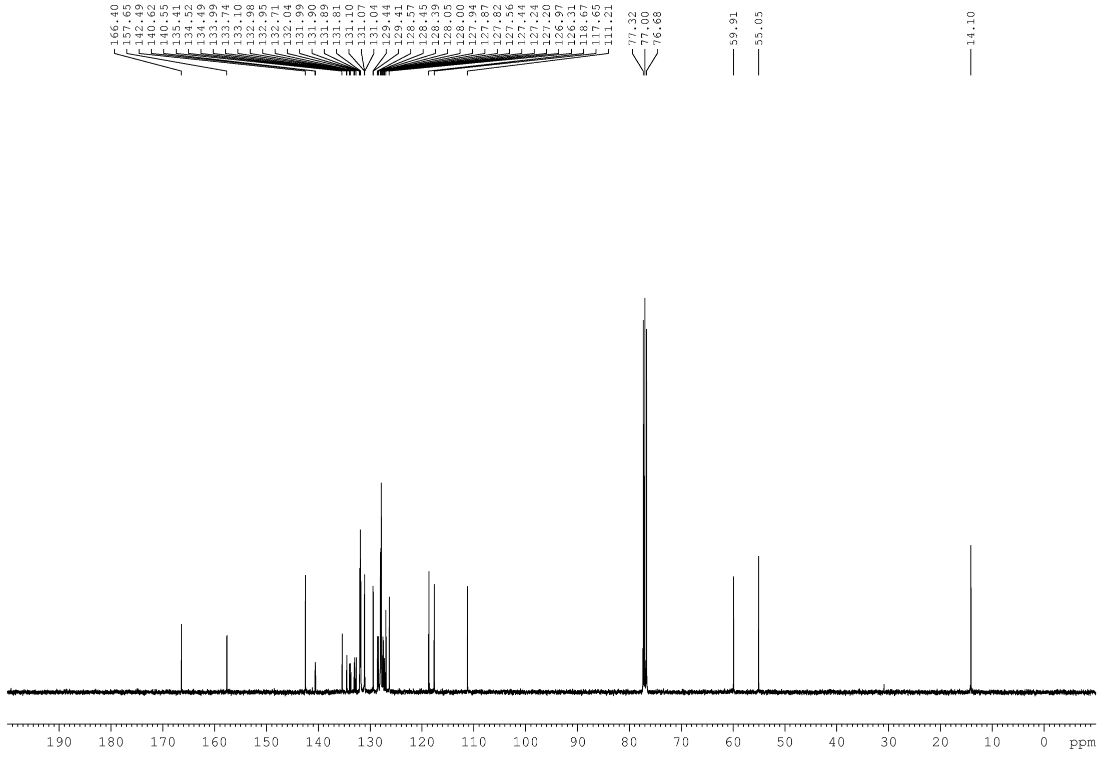
166.40 157.65 142.49 140.62 140.55 135.41 134.52 134.49 133.99 133.74 133.10 132.98 132.95 132.71 132.04 131.99 131.90 131.89 131.81 131.10 131.07 131.04 129.44 129.41 128.57 128.45 128.39 128.05 128.00 127.94 127.87 127.82 127.56 127.44 127.24 127.20 126.97 126.31 118.67 117.65 111.21
77.32 77.00 76.68
59.91
55.05
14.10
190 180 170 160 150 140 130 120 110 100 90 80 70 60 50 40 30 20 10 0 ppm

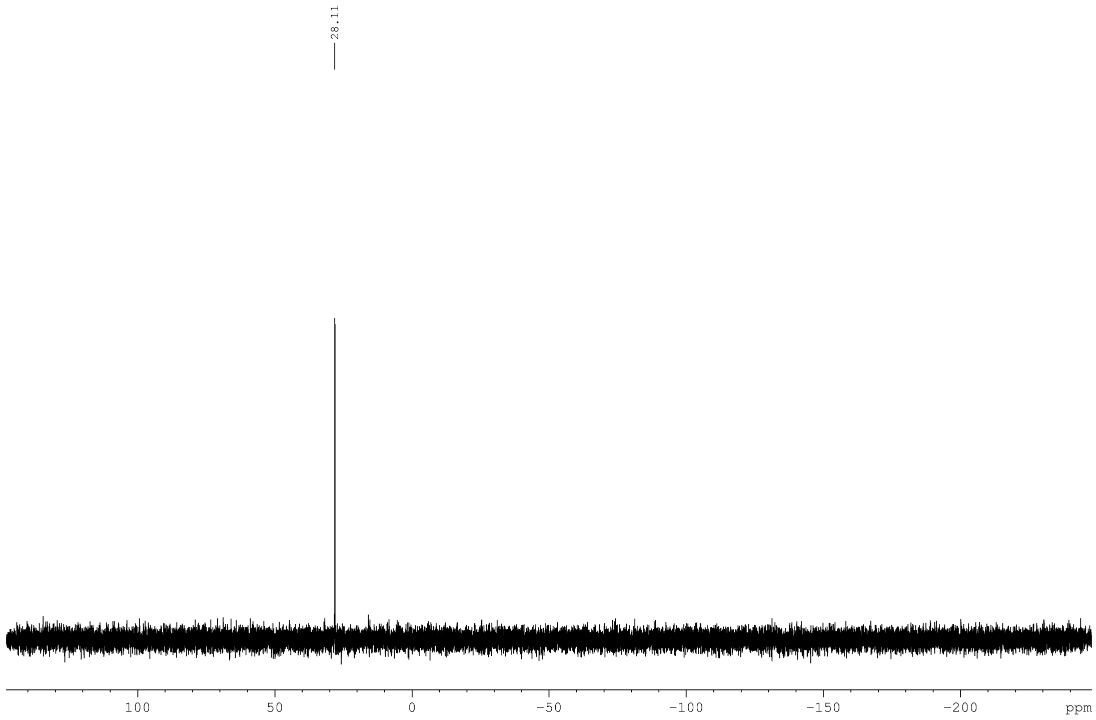

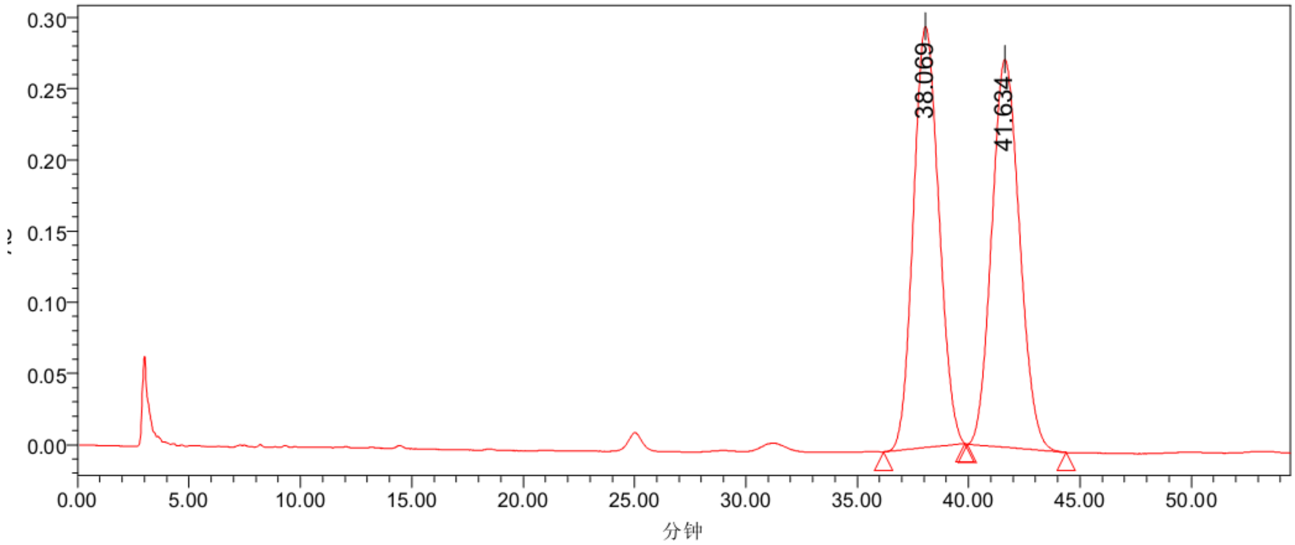

处理通道: **PDA 229.7 纳米**

	处理通道	保留时间 (分钟)	面积	% 面积	峰高
1	PDA 229.7 纳米	38.069	23460782	49.75	295552
2	PDA 229.7 纳米	41.634	23695855	50.25	272443

Processing channel: PDA 229.7nm

	Processing channel	Retention time (minute)	Area	Area %	Peak height
1	PDA 229.7nm	38.069	23460782	49.75	295552
2	PDA 229.7nm	41.634	23695855	50.25	272443

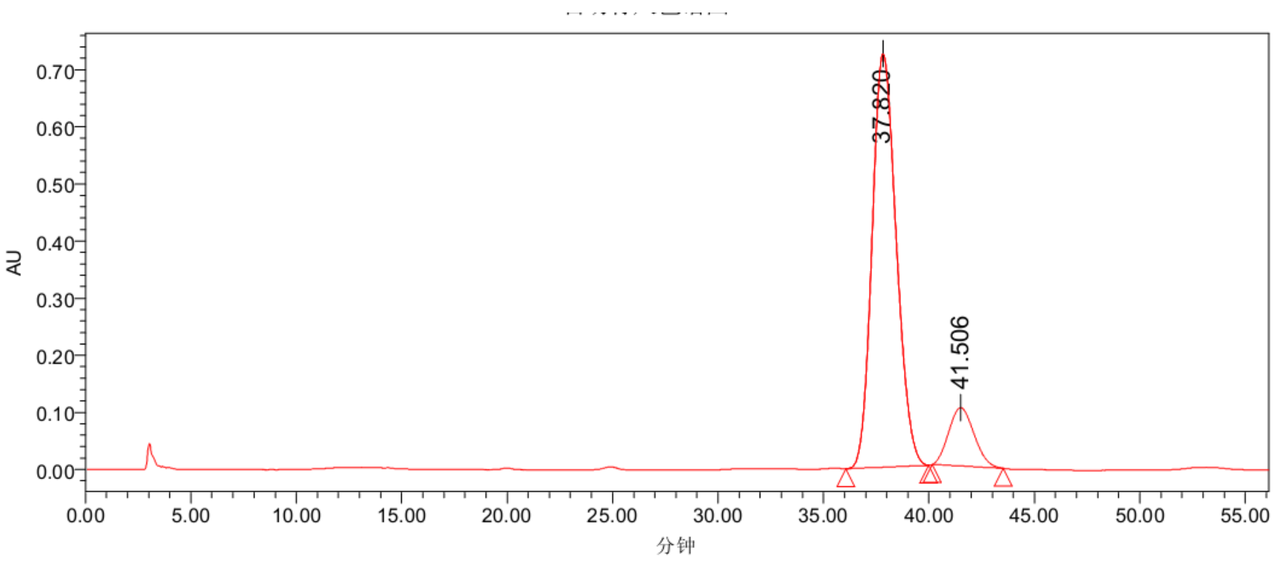

处理通道: PDA 235.1 纳米

	处理通道	保留时间 (分钟)	面积	% 面积	峰高
1	PDA 235.1 纳米	37.820	57678578	87.07	724170
2	PDA 235.1 纳米	41.506	8564760	12.93	102220

Processing channel: PDA 235.1nm

	Processing channel	Retention time (minute)	Area	Area %	Peak height
1	PDA 235.1nm	37.820	57678578	87.07	724170
2	PDA 235.1nm	41.506	8560760	12.93	102220

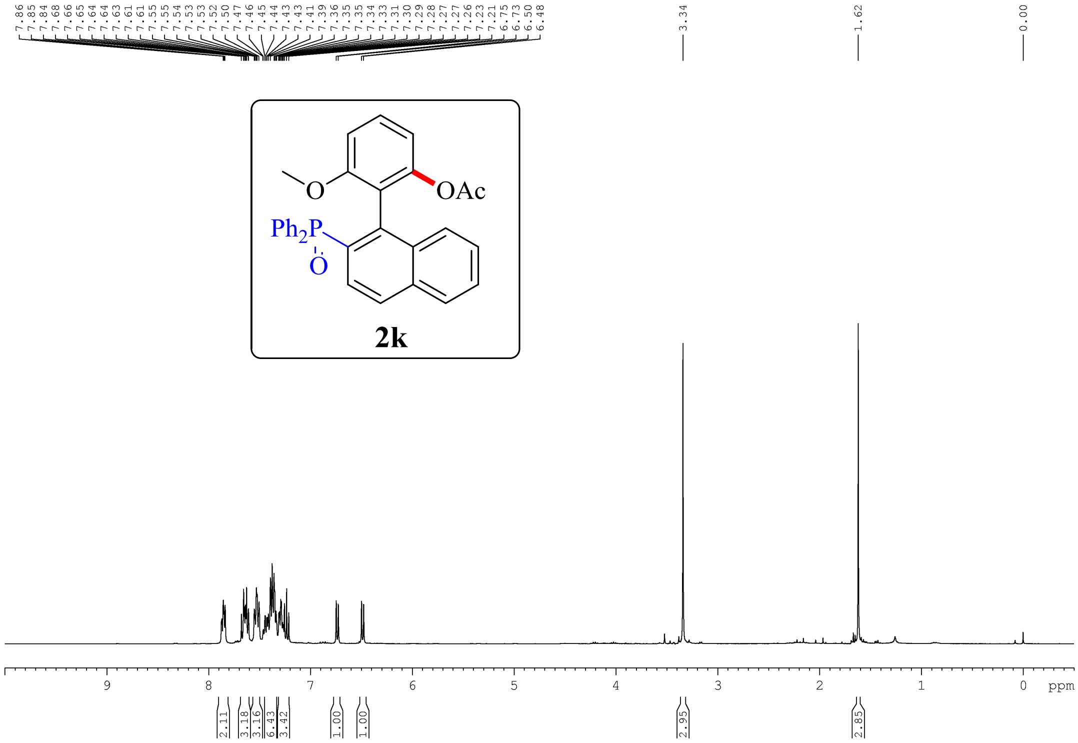
7.86 7.85 7.84 7.68 7.66 7.65 7.64 7.64 7.63 7.61 7.61 7.55 7.55 7.54 7.53 7.53 7.52 7.50 7.47 7.46 7.45 7.44 7.43 7.43 7.41 7.39 7.36 7.35 7.35 7.34 7.33 7.31 7.30 7.29 7.28 7.27 7.27 7.26 7.23 7.21 6.75 6.73 6.50 6.48
3.34
1.62
0.00
O
OAc
Ph2P
O
2k
9 8 7 6 5 4 3 2 1 0 ppm
2.11 3.18 3.16 6.43 3.42 1.00 1.00 2.95 2.85

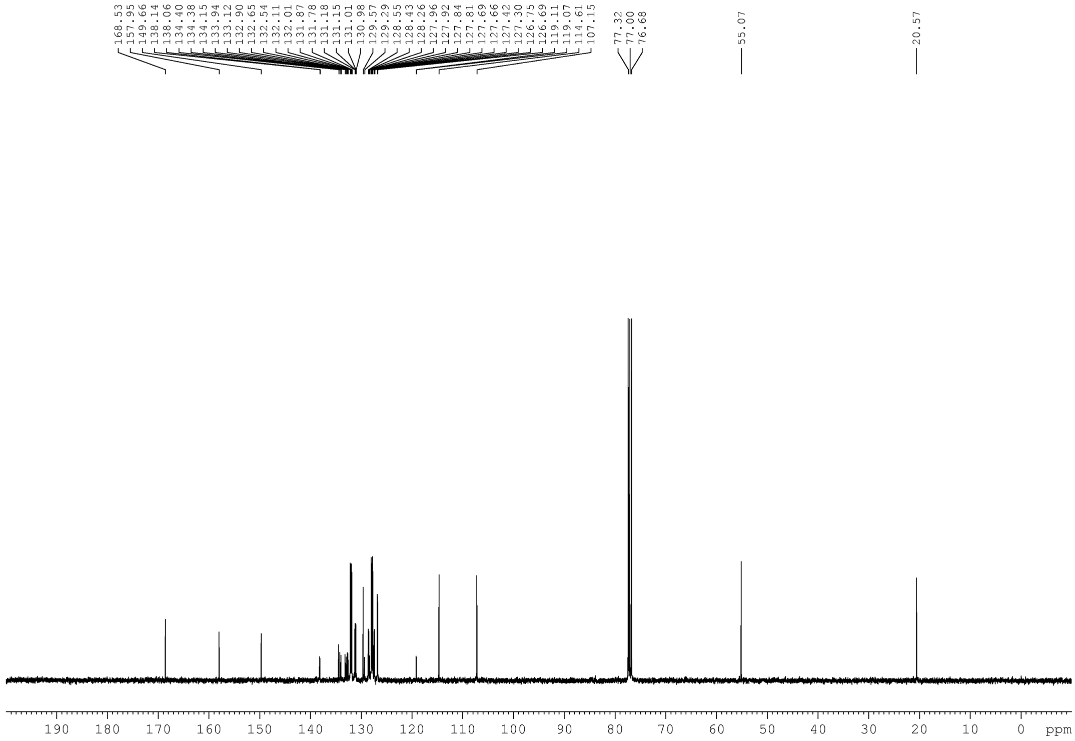
168.53 157.95 149.66 138.14 138.06 134.40 134.38 134.15 133.94 133.12 132.90 132.65 132.54 132.11 132.01 131.87 131.78 131.18 131.15 131.01 130.98 129.57 129.29 128.55 128.43 128.26 127.96 127.92 127.84 127.81 127.69 127.66 127.42 127.30 126.75 126.69 119.11 119.07 114.61 107.15
77.32 77.00 76.68
55.07
20.57
190 180 170 160 150 140 130 120 110 100 90 80 70 60 50 40 30 20 10 0 ppm

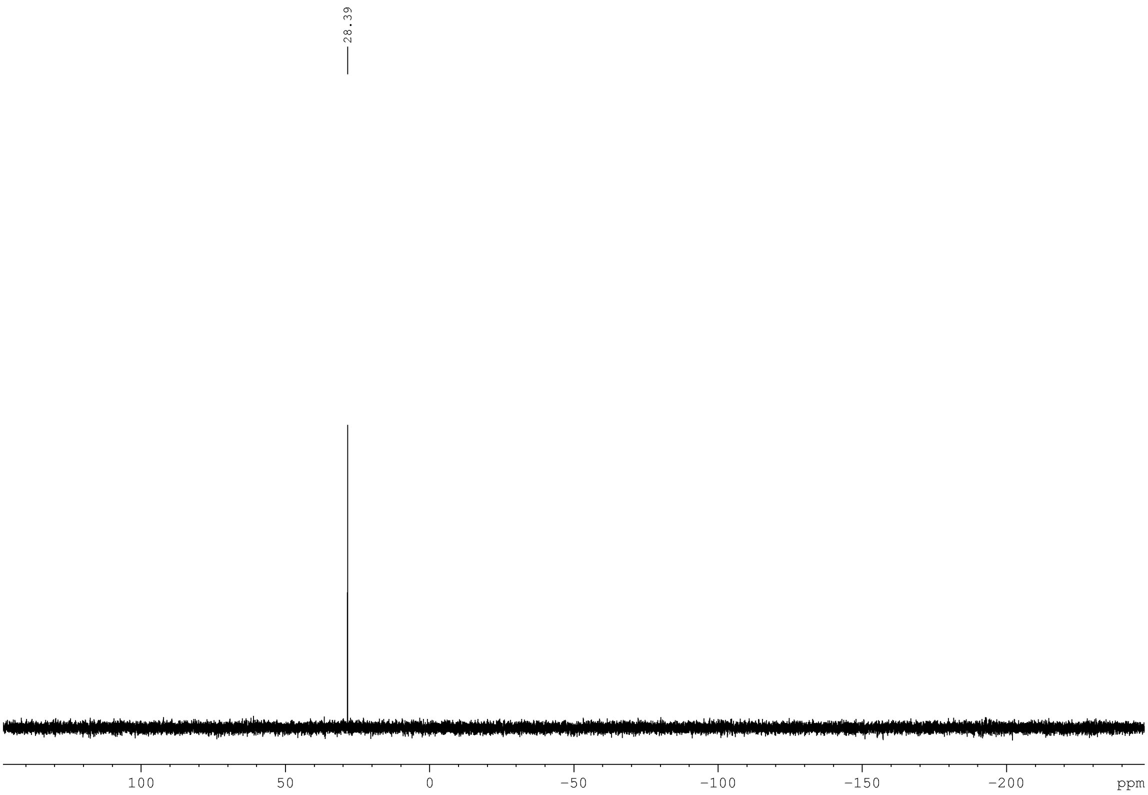

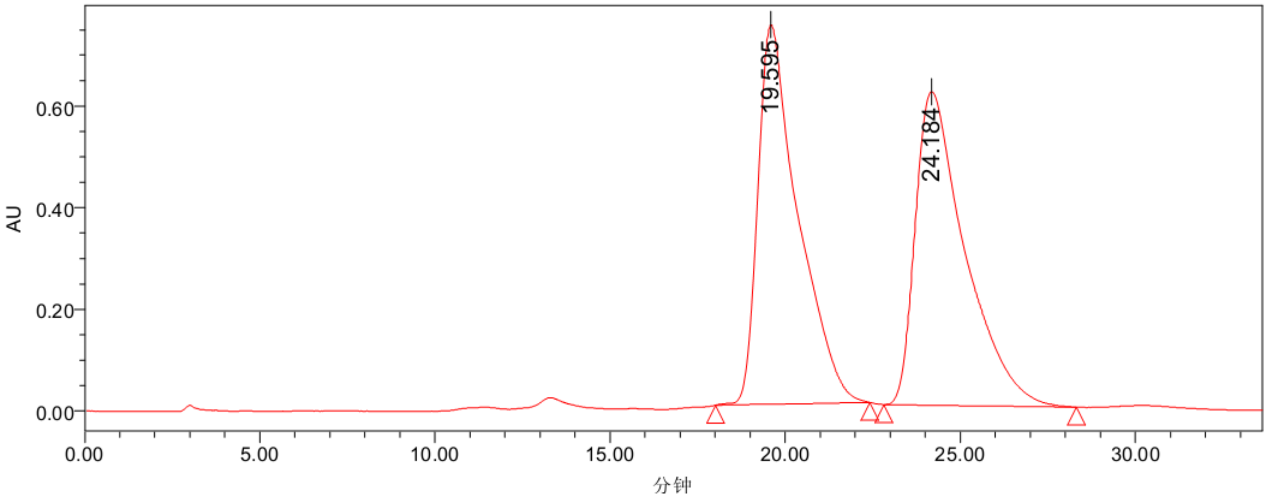

处理通道: PDA 236.7 纳米

	处理通道	保留时间 (分钟)	面积	% 面积	峰高
1	PDA 236.7 纳米	19.595	59648584	49.45	747000
2	PDA 236.7 纳米	24.184	60979640	50.55	617442

Processing channel: PDA 236.7nm

	Processing channel	Retention time (minute)	Area	Area %	Peak height
1	PDA 236.7nm	19.595	59648584	49.45	747000
2	PDA 236.7nm	24.184	60979640	50.55	617442

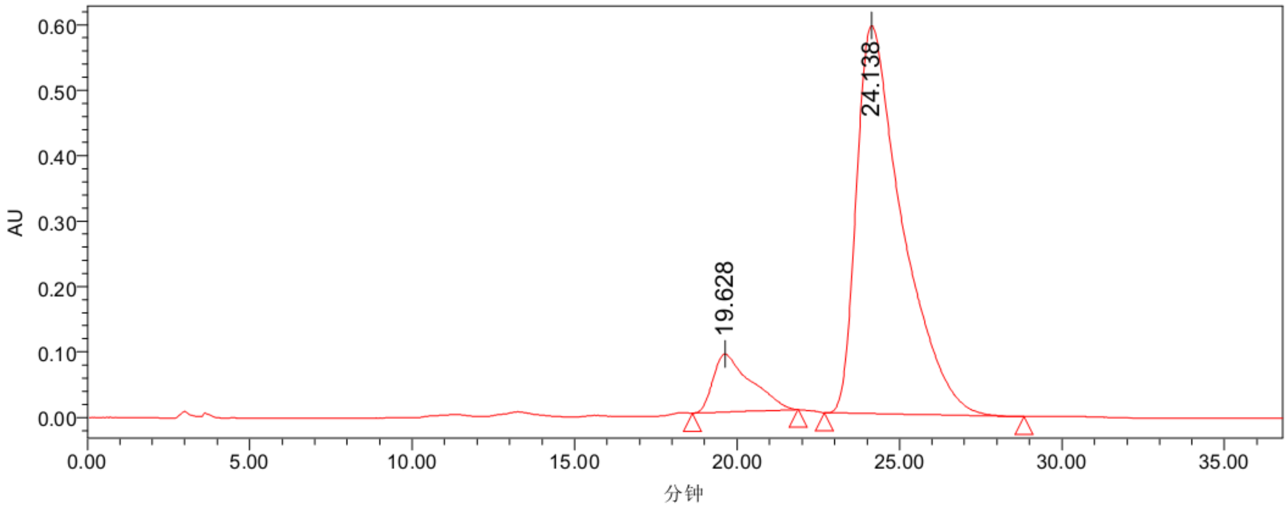

处理通道: PDA 236.7 纳米

	处理通道	保留时间 (分钟)	面积	% 面积	峰高
1	PDA 236.7 纳米	19.628	7123913	10.88	88652
2	PDA 236.7 纳米	24.138	58329832	89.12	593536

Processing channel: PDA 236.7nm

	Processing channel	Retention time (minute)	Area	Area %	Peak height
1	PDA 236.7nm	19.628	7123913	10.88	88652
2	PDA 236.7nm	24.138	58329832	89.12	593536

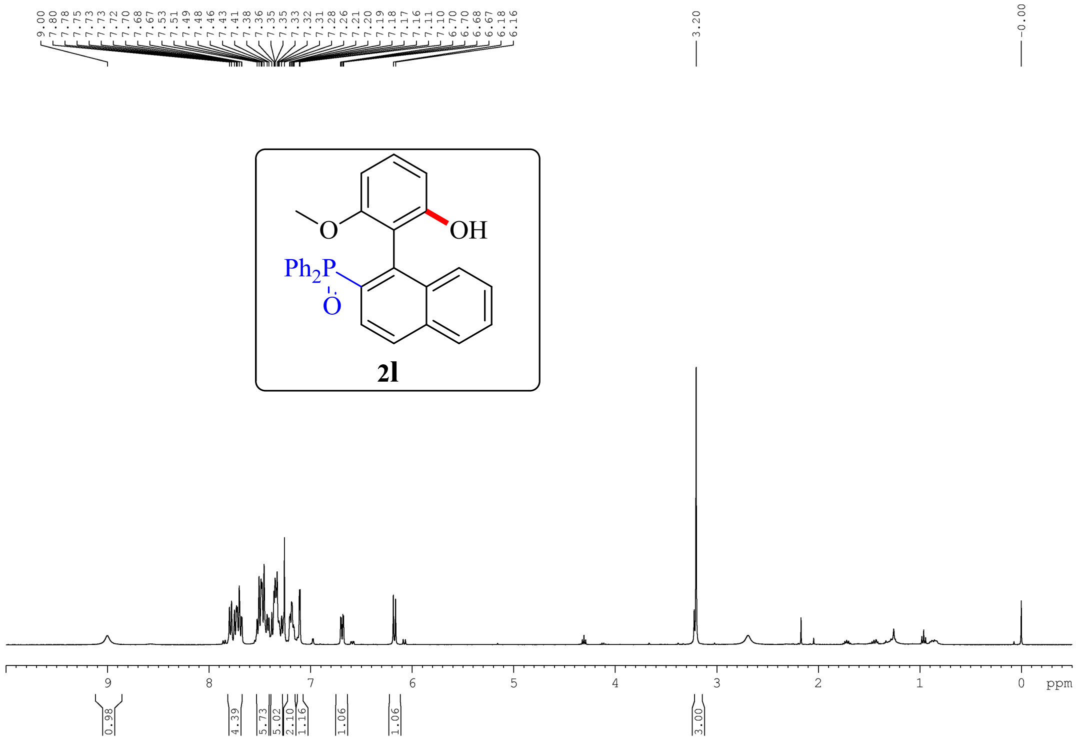
9.00 7.80 7.78 7.75 7.73 7.73 7.72 7.70 7.68 7.67 7.53 7.51 7.49 7.48 7.46 7.43 7.41 7.38 7.36 7.35 7.35 7.33 7.32 7.31 7.28 7.26 7.21 7.20 7.19 7.18 7.17 7.16 7.11 7.10 6.70 6.70 6.68 6.67 6.18 6.16
3.20
-0.00
O
OH
Ph2P
O
2l
9 8 7 6 5 4 3 2 1 0 ppm
0.98 4.39 5.73 5.02 2.10 1.16 1.06 1.06 3.00

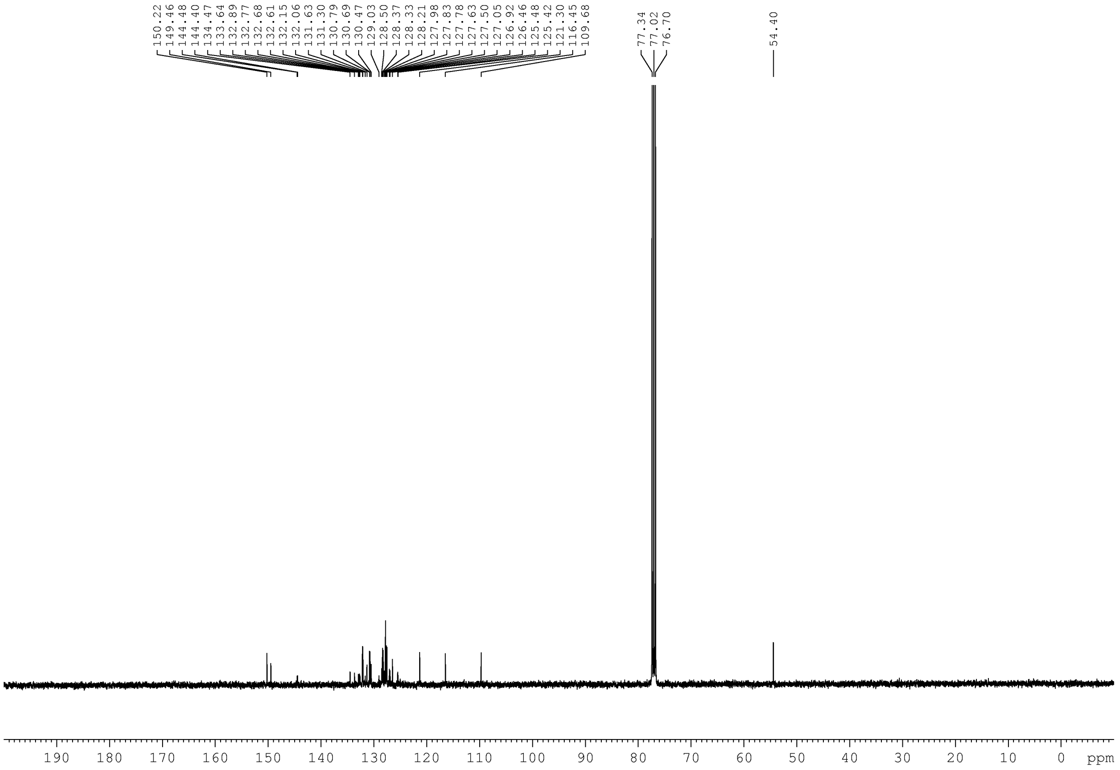
150.22 149.46 144.48 144.40 134.47 133.64 132.89 132.77 132.68 132.61 132.15 132.06 131.63 131.30 130.79 130.69 130.47 129.03 128.50 128.37 128.33 128.21 127.98 127.83 127.78 127.63 127.50 127.05 126.92 126.46 125.48 125.42 121.30 116.45 109.68
77.34 77.02 76.70
54.40
190 180 170 160 150 140 130 120 110 100 90 80 70 60 50 40 30 20 10 0 ppm

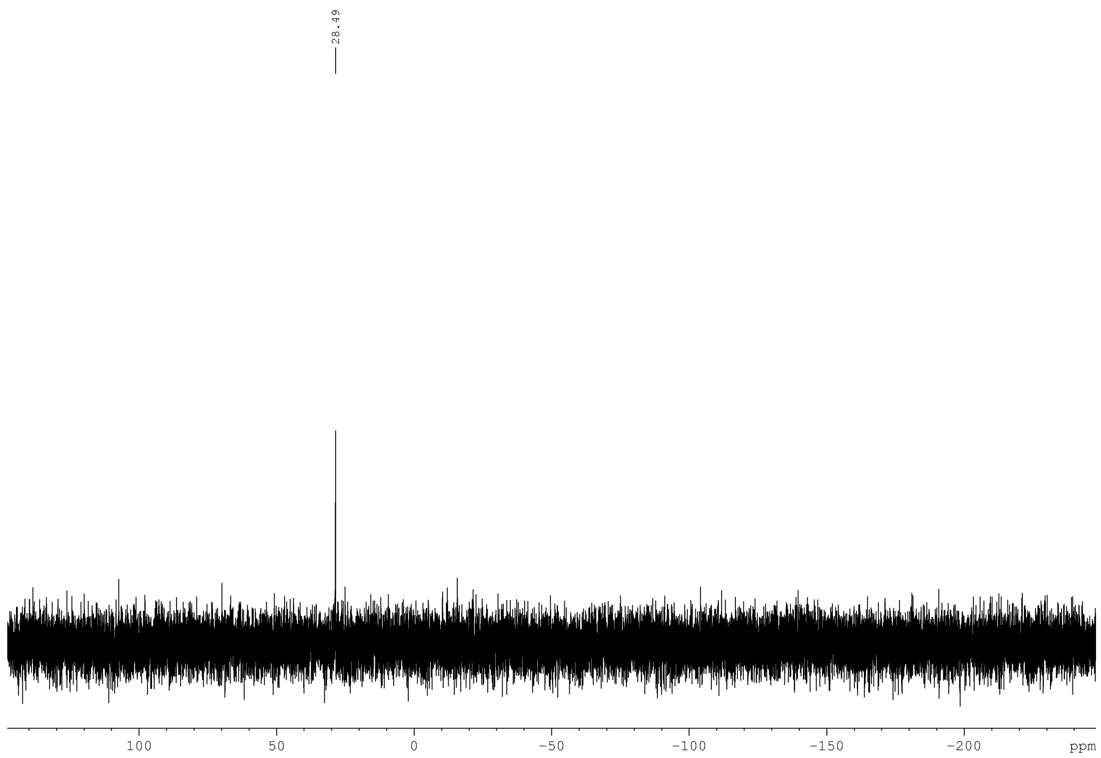

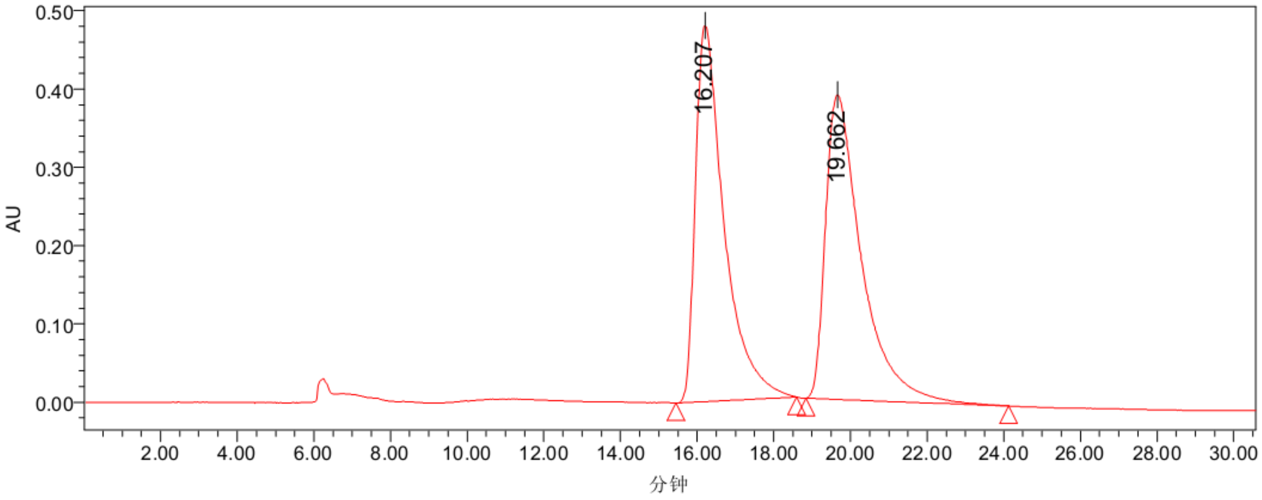

处理通道: PDA 237.9 纳米

	处理通道	保留时间 (分钟)	面积	% 面积	峰高
1	PDA 237.9 纳米	16.207	24960109	49.63	480433
2	PDA 237.9 纳米	19.662	25331296	50.37	389016

Processing channel: PDA 237.9nm

	Processing channel	Retention time (minute)	Area	Area %	Peak height
1	PDA 237.9nm	16.207	24960109	49.63	480433
2	PDA 237.9nm	19.662	25331296	50.37	389016

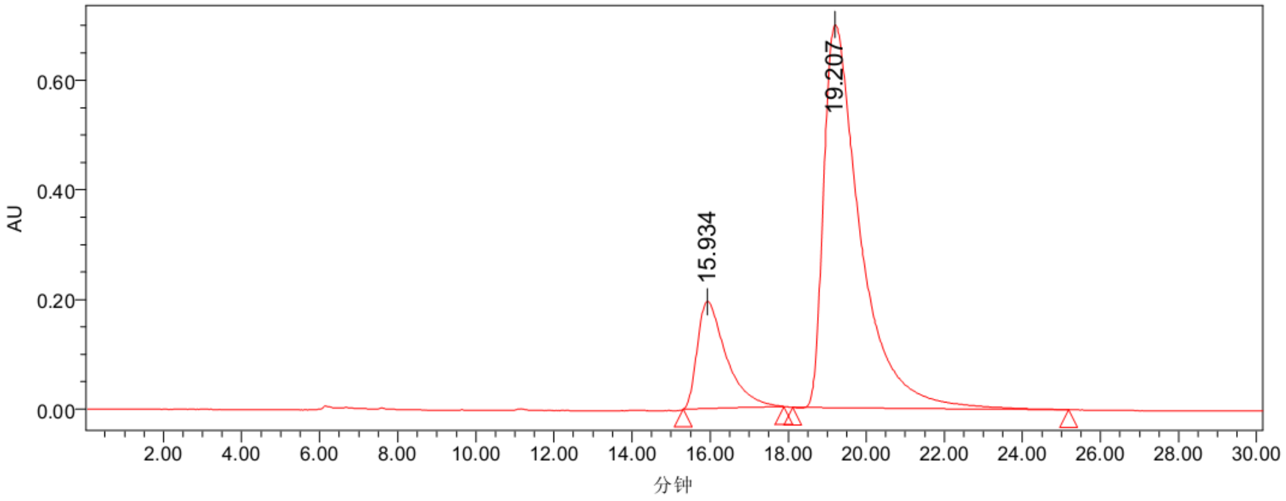

处理通道: PDA 237.9 纳米

	处理通道	保留时间 (分钟)	面积	% 面积	峰高
1	PDA 237.9 纳米	15.934	10061404	18.05	194948
2	PDA 237.9 纳米	19.207	45676134	81.95	699301

Processing channel: PDA 237.9nm

	Processing channel	Retention time (minute)	Area	Area %	Peak height
1	PDA 237.9nm	15.934	10061404	18.05	194948
2	PDA 237.9nm	19.207	45676134	81.95	699301

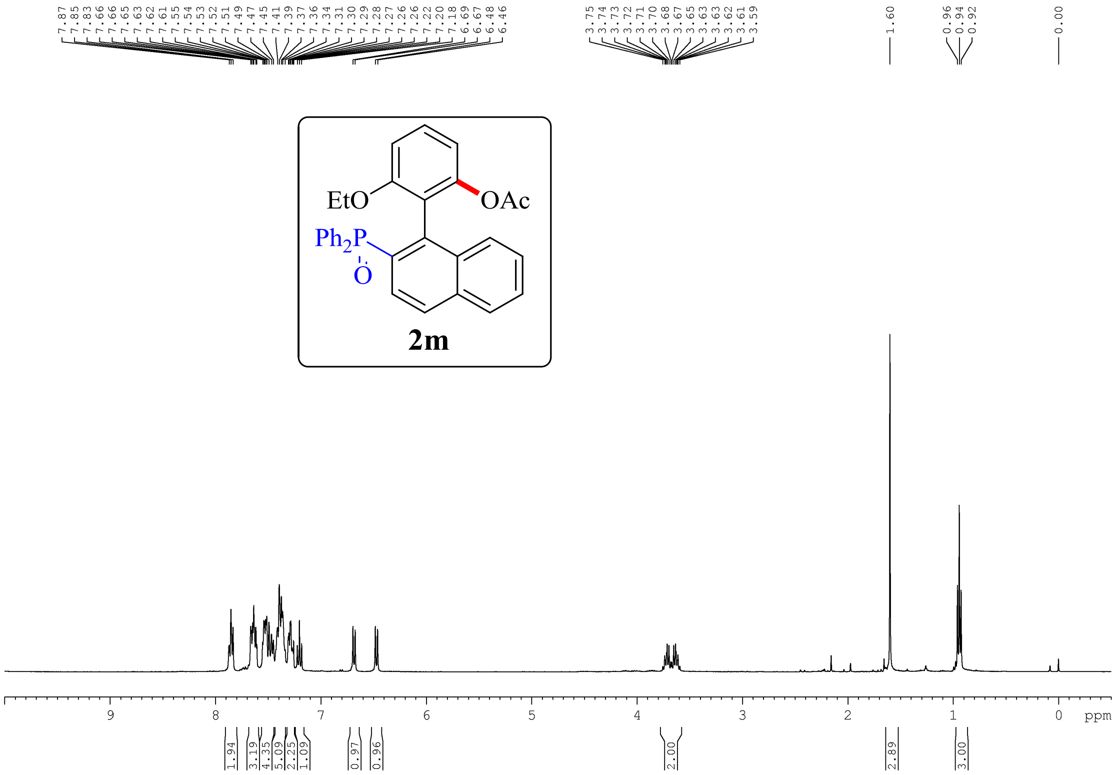
7.87
7.85
7.83
7.66
7.66
7.65
7.63
7.62
7.61
7.55
7.54
7.53
7.52
7.51
7.49
7.47
7.45
7.41
7.39
7.37
7.36
7.34
7.31
7.30
7.29
7.28
7.27
7.26
7.22
7.20
7.18
6.69
6.67
6.48
6.46
3.75
3.74
3.73
3.72
3.71
3.70
3.68
3.67
3.65
3.63
3.63
3.62
3.61
3.59
1.60
0.96
0.94
0.92
0.00
EtO
OAc
Ph_2P
O
2m
9
8
7
6
5
4
3
2
1
0
ppm
1.94
3.19
4.35
5.09
2.25
1.09
0.97
0.96
2.00
2.89
3.00

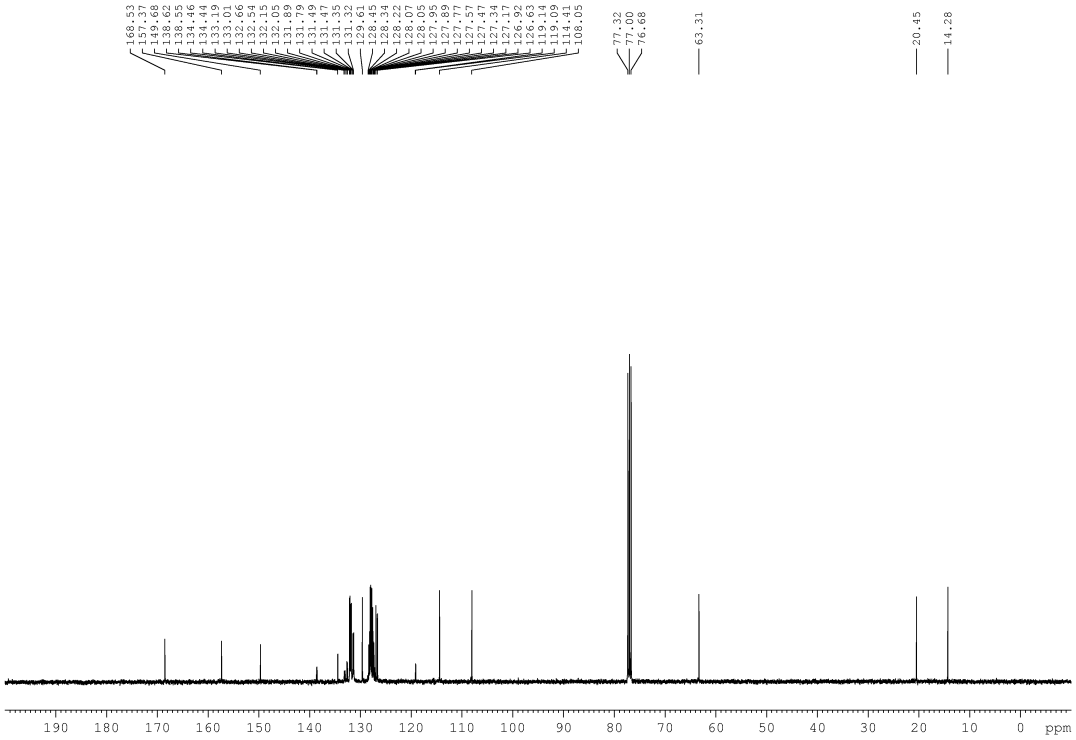
168.53
157.37
149.68
138.62
138.55
134.46
134.44
133.19
133.01
132.66
132.54
132.15
132.05
131.89
131.79
131.49
131.47
131.35
131.32
129.61
128.45
128.34
128.22
128.07
128.05
127.95
127.89
127.77
127.57
127.47
127.34
127.17
126.92
126.63
119.14
119.09
114.41
108.05
77.32
77.00
76.68
63.31
20.45
14.28
190
180
170
160
150
140
130
120
110
100
90
80
70
60
50
40
30
20
10
0
ppm

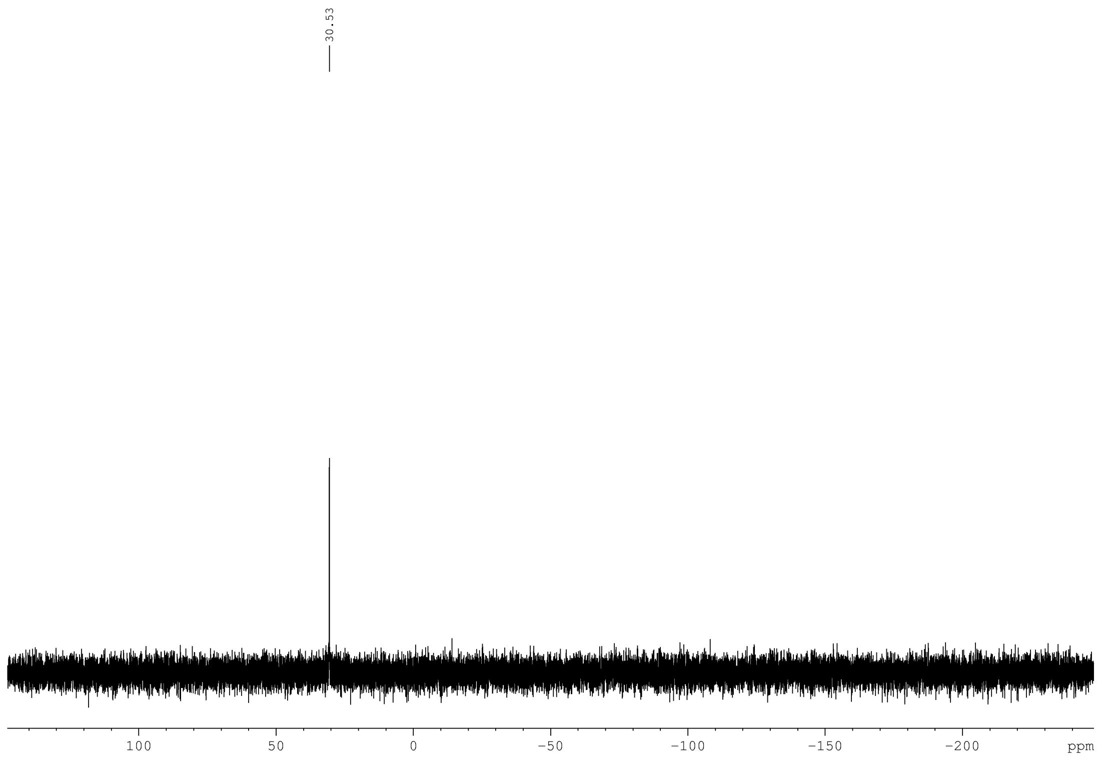

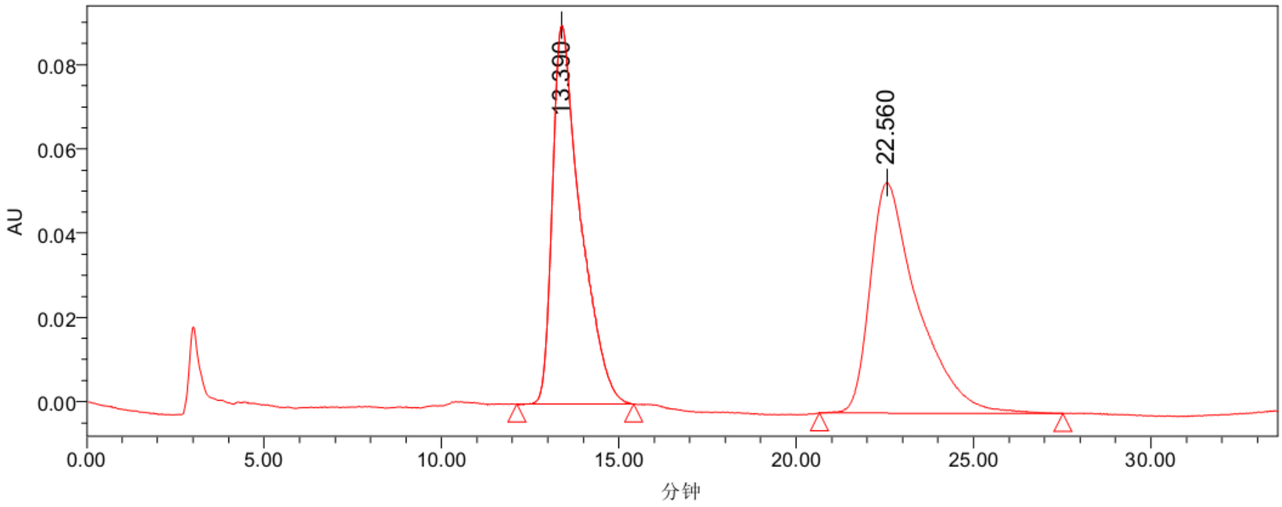

处理通道: PDA 236.7 纳米

	处理通道	保留时间 (分钟)	面积	% 面积	峰高
1	PDA 236.7 纳米	13.390	4914848	49.13	89950
2	PDA 236.7 纳米	22.560	5088822	50.87	54735

Processing channel: PDA 236.7nm

	Processing channel	Retention time (minute)	Area	Area %	Peak height
1	PDA 236.7nm	13.390	4914848	49.13	89950
2	PDA 236.7nm	22.560	5088822	50.87	54735

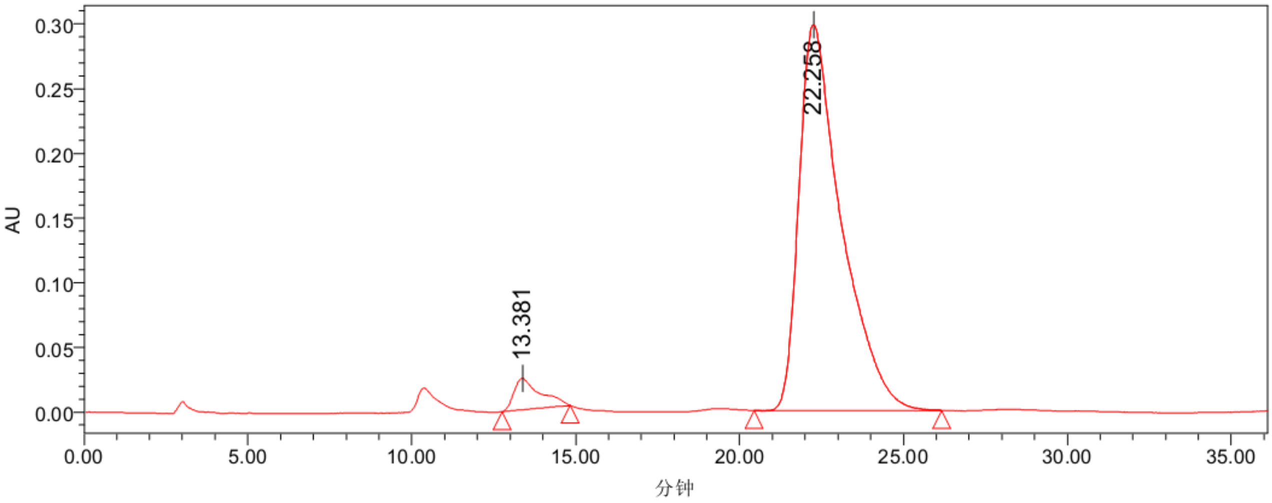

处理通道: **PDA 236.7** 纳米

	处理通道	保留时间 (分钟)	面积	% 面积	峰高
1	PDA 236.7 纳米	13.381	1353855	4.80	24141
2	PDA 236.7 纳米	22.258	26825678	95.20	298324

Processing channel: PDA 236.7nm

	Processing channel	Retention time (minute)	Area	Area %	Peak height
1	PDA 236.7nm	13.381	1353855	4.80	24141
2	PDA 236.7nm	22.258	26825678	95.20	298324

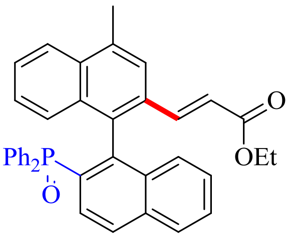

2i

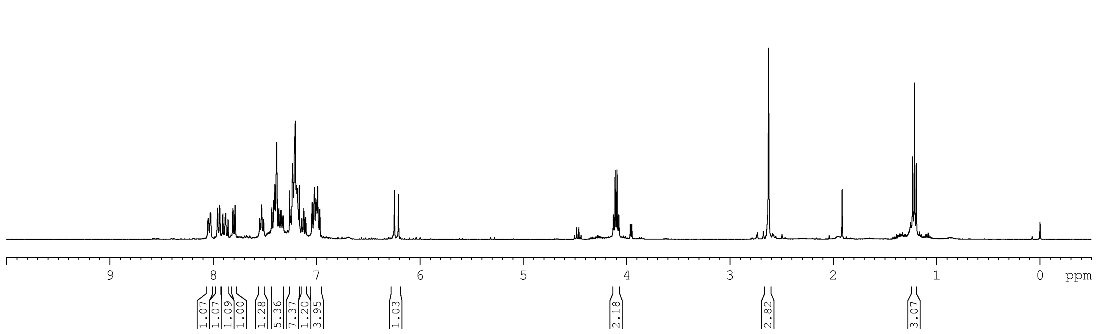

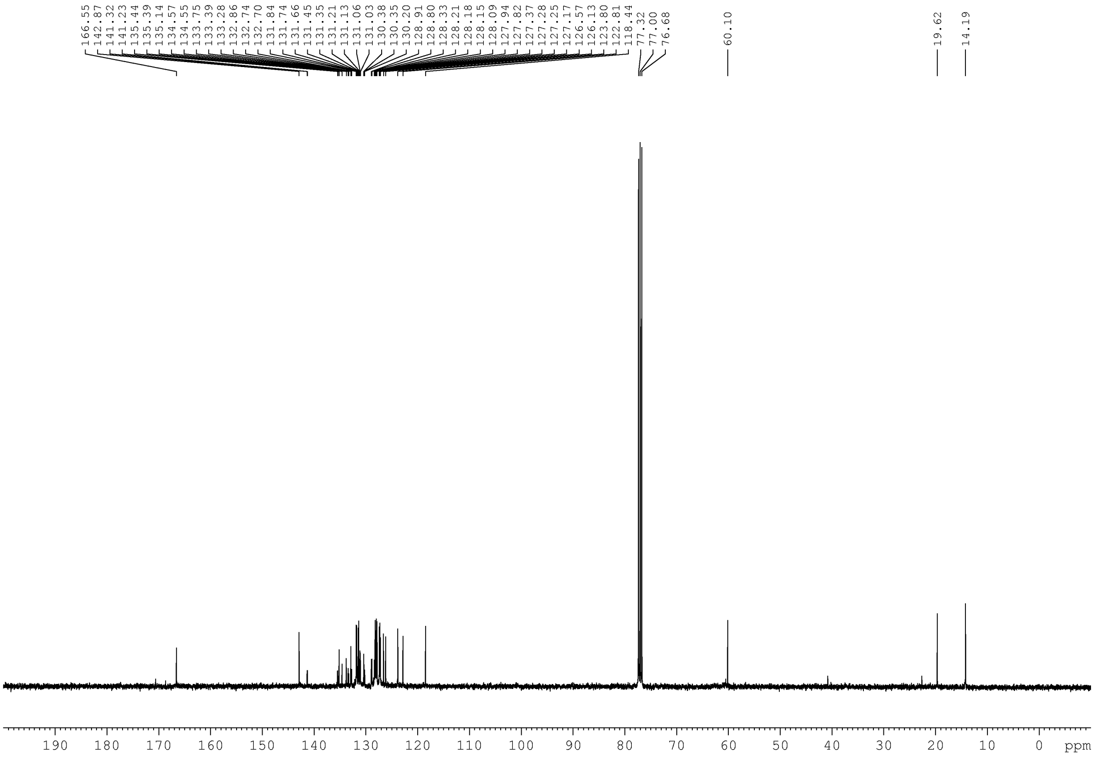

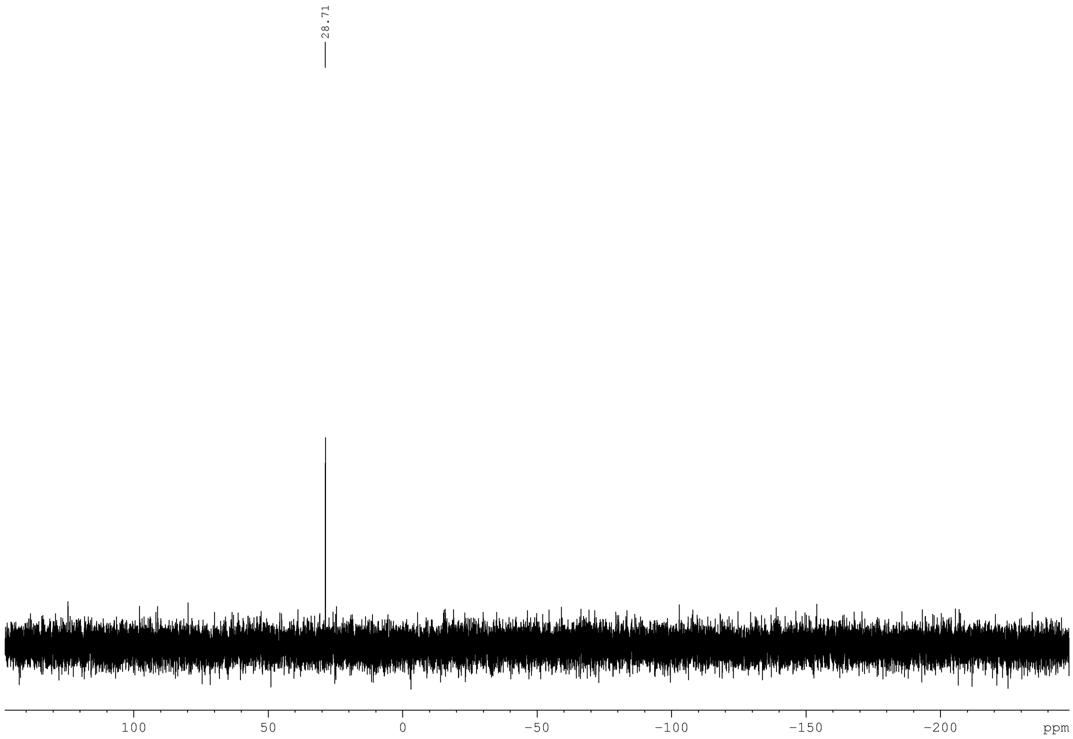
28.71
100
50
0
-50
-100
-150
-200
ppm

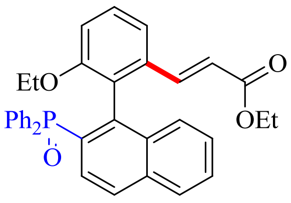
EtO
Ph_2P
O
O
OEt

2n

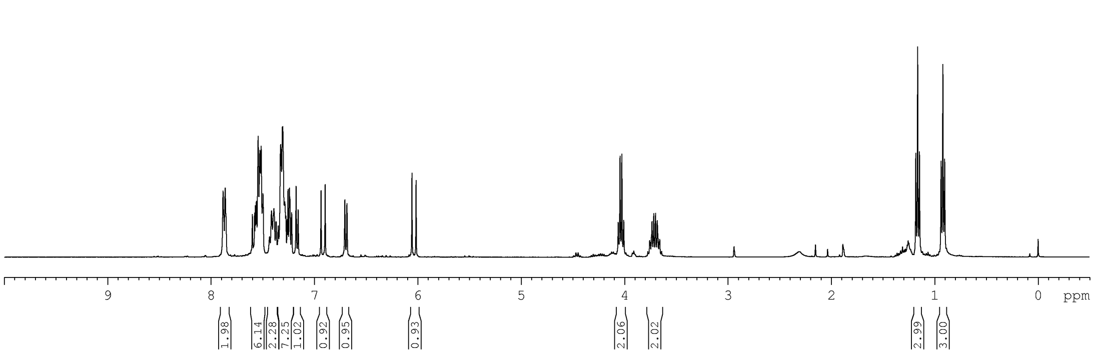
7.88 7.86 7.60 7.58 7.57 7.56 7.54 7.52 7.52 7.51 7.50 7.50 7.43 7.41 7.39 7.37 7.37 7.35 7.32 7.31 7.30 7.28 7.27 7.26 7.25 7.24 7.23 7.22 7.17 7.15 6.93 6.89 6.70 6.68 6.05 6.01
4.06 4.04 4.02 4.01 3.77 3.75 3.75 3.73 3.71 3.70 3.68 3.66 3.64
1.18 1.17 1.15 0.94 0.92 0.90
0.00
9
8
7
6
5
4
3
2
1
0
ppm
1.98 6.14 2.28 7.25 1.02 0.92 0.95 0.93 2.06 2.02 2.99 3.00

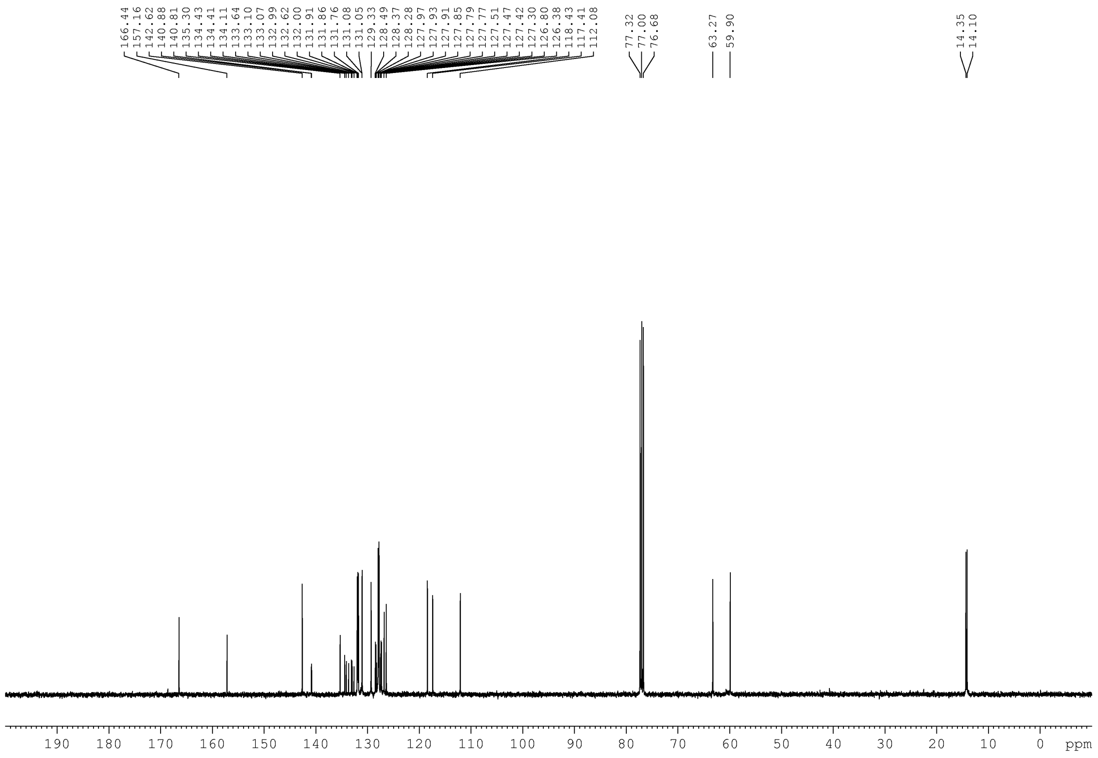
166.44
157.16
142.62
140.88
140.81
135.30
134.43
134.41
134.11
133.64
133.10
133.07
132.99
132.62
132.00
131.91
131.86
131.76
131.08
131.05
129.33
128.49
128.37
128.28
127.97
127.93
127.91
127.85
127.79
127.77
127.51
127.47
127.42
127.30
126.80
126.38
118.43
117.41
112.08
77.32
77.00
76.68
63.27
59.90
14.35
14.10
190 180 170 160 150 140 130 120 110 100 90 80 70 60 50 40 30 20 10 0 ppm

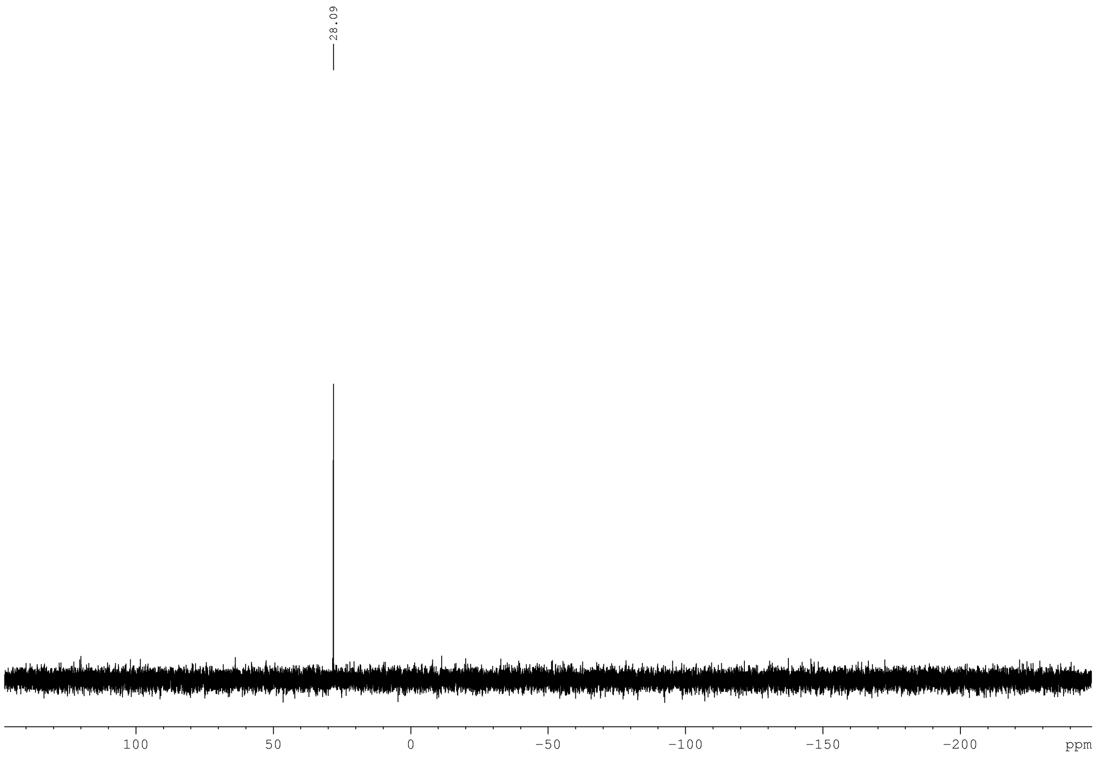
28.09
100 50 0 −50 −100 −150 −200 ppm

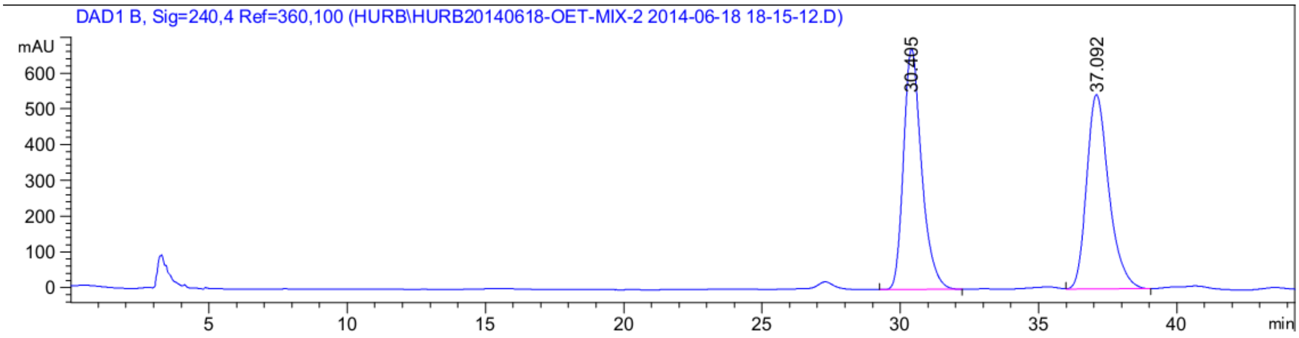

峰 #	保留时间 [min]	类型	峰宽 [min]	峰面积 [mAU*s]	峰高 [mAU]	峰面积 %
1	30.405	BB	0.6932	3.06682e4	673.51056	50.2668
2	37.092	VB	0.8535	3.03427e4	543.38837	49.7332

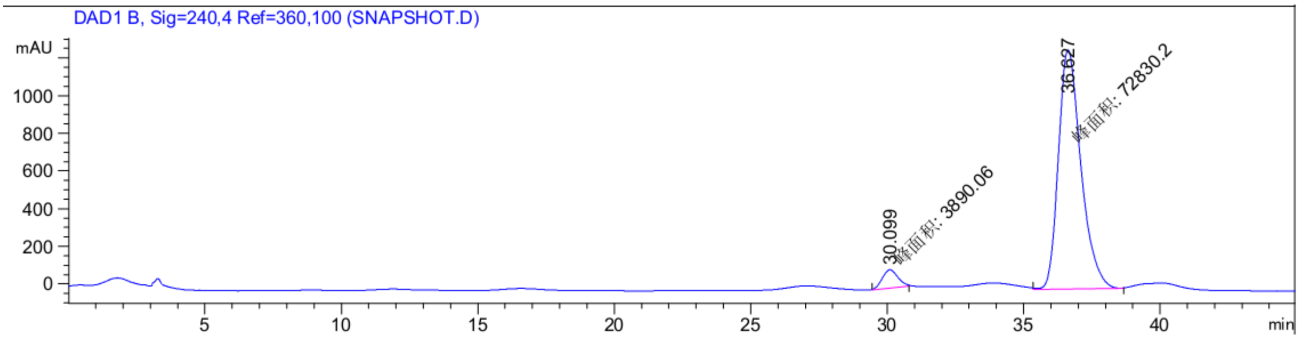

峰 #	保留时间 [min]	类型	峰宽 [min]	峰面积 [mAU*s]	峰高 [mAU]	峰面积 %
1	30.099	MM	0.6683	3890.06152	97.00778	5.0705
2	36.627	MM	0.9569	7.28302e4	1268.47192	94.9295
